# Minimal physiologically‐based hybrid model of pharmacokinetics in pregnant women: Application to antenatal corticosteroids

**DOI:** 10.1002/psp4.12899

**Published:** 2023-03-14

**Authors:** Wojciech Krzyzanski, Mark A. Milad, Alan H. Jobe, William J. Jusko

**Affiliations:** ^1^ School of Pharmacy and Pharmaceutical Sciences, State University of New York University of Buffalo Buffalo New York USA; ^2^ Milad Pharmaceutical Consulting LLC Plymouth Michigan USA; ^3^ Division of Pulmonary Biology Cincinnati Children's Hospital Medical Center, University of Cincinnati Cincinnati Ohio USA

## Abstract

Minimal physiologically‐based pharmacokinetic (mPBPK) models are an alternative to full physiologically‐based pharmacokinetic (PBPK) models as they offer reduced complexity while maintaining the physiological interpretation of key model components. Full PBPK models have been developed for pregnancy, but a mPBPK model eases the ability to perform a “top‐down” meta‐analysis melding all available pharmacokinetic (PK) data in the mother and fetus. Our hybrid mPBPK model consists of mPBPK models for the mother and fetus with connection by the placenta. This model was applied to describe the rich PK data of antenatal corticosteroid betamethasone (BET) jointly with the limited data for dexamethasone (DEX) in the mother and fetus. Physiologic model parameters were obtained from the literature while drug‐dependent parameters were estimated by the simultaneous fitting of all available data for DEX and BET. Maternal clearances of DEX and BET confirmed the literature values, and the expected fetal‐to‐maternal plasma ratios ranged from 0.3 to 0.4 for both drugs. Simulations of maternal plasma concentrations for the dosing regimens of BET and DEX recommended by the World Health Organization based on our findings revealed up to 60% lower exposures than found in nonpregnant women and offers a means of devising alternative dosing regimens. Our hybrid mPBPK model and meta‐analysis approach could facilitate assessment of other classes of drugs indicated for the treatment of pregnant women.

**Study Highlights**

**WHAT IS THE CURRENT KNOWLEDGE ON THE TOPIC?**

Minimal physiologically‐based pharmacokinetic (mPBPK) models are less complex than physiologically‐based pharmacokinetic (PBPK) models but still maintain the physiological interpretation of key model components. PBPK models have been developed for pregnancy but offer complexities in the analysis of typical pharmacokinetic (PK) data from multiple studies.

**WHAT QUESTION DID THIS STUDY ADDRESS?**

Can hybrid mPBPK models serve as an alternative to full PBPK models of drugs in pregnancy in assessing multiple sources of PK data?

**WHAT DOES THIS STUDY ADD TO OUR KNOWLEDGE?**

Hybrid mPBPK models of drugs in pregnant women allow the meta‐analysis of multiple sources of PK data to improve quantitation and prediction of plasma drug concentrations in the mother and fetus. The study predicts the concentration time profiles of dexamethasone and betamethasone in the fetus during antenatal treatment, typically unobtainable using traditional PK sampling.

**HOW MIGHT THIS CHANGE DRUG DISCOVERY, DEVELOPMENT, AND/OR THERAPEUTICS?**

Our hybrid mPBPK model allows for in silico testing of corticosteroid dosing regimens to help evaluate expected PK profiles with respect to potential efficacy and adverse effects in the fetus.

## INTRODUCTION

Modeling drug pharmacokinetics (PK) in pregnant women is challenging due to dynamic changes in many physiological and biochemical functions that remain otherwise constant. The presence of the fetus with a limited accessibility for blood sampling creates additional complexity. These challenges fall into the scope of physiologically‐based PK (PBPK) models that can be designed to describe such dynamic and complex systems. The PBPK model structure and level of complexity are commonly determined by application to specific drugs. There has been a steady increase in development of PBPK models to account for changes in disposition during pregnancy. Such PBPK models have been developed for antibiotics,^1^ antivirals,^2^ analgesics,^3^ corticosteroids (CSs),^4^ and nonsteroidal anti‐inflammatory drugs.^5^ Application of PBPK models can provide guidance on drug dosing during pregnancy.^6^

PBPK models involve many organs, tissues, and processes moving drug molecules through the body. Each organ and tissue can be further divided into subcomponents. This granularity is reflected in the complex model structure and number of model parameters that need to be determined from external sources or available data. Organ lumping has been enacted to reduce the complexity of PBPK models.^7^ Minimal PBPK (mPBPK) models offer a consolidation approach where only two or three groups of organs and tissues represent the whole body.^8^ Blood flow through the grouped organs is partitioned using fractions of the cardiac output, and organ weights/volumes add up to the body mass. Such mPBPK models facilitate a top‐down assessment of multiple sources of PK data as demonstrated by our meta‐analyses of dexamethasone (DEX) PK across 11 species.^9^ To our knowledge, a mPBPK model representing pregnancy has not been yet developed for any drug. A full PBPK model for BET and DEX in pregnancy has been introduced^4^ (as will be discussed).

Respiratory distress syndrome (RDS) is the most significant factor contributing to morbidity and mortality of prematurely born neonates. High‐dose antenatal CSs are the standard treatment of women at risk of preterm delivery.^10^ Prolonged exposure to antenatal CSs can cause severe adverse effects in the fetus.^11^ Current recommended treatments by the World Health Organization (WHO) comprise dexamethasone phosphate (DEX‐P) or betamethasone phosphate (BET‐P) given via intramuscular (i.m.) administration.^12^ The therapeutic window for fetal CS plasma concentrations is not known.

One objective of this report is to develop a general hybrid mPBPK model for drugs in pregnancy. The developed mPBPK model was applied to describe the PK of antenatal CSs in the mother and fetus using all available literature data from diverse and rich sources for betamethasone (BET) and limited data for DEX. Finally, we used our hybrid mPBPK model to simulate maternal and fetal CS plasma concentrations for the therapeutic dosing regimens recommended by the WHO.

## METHODS

### mPBPK model in pregnancy

**FIGURE 1:**
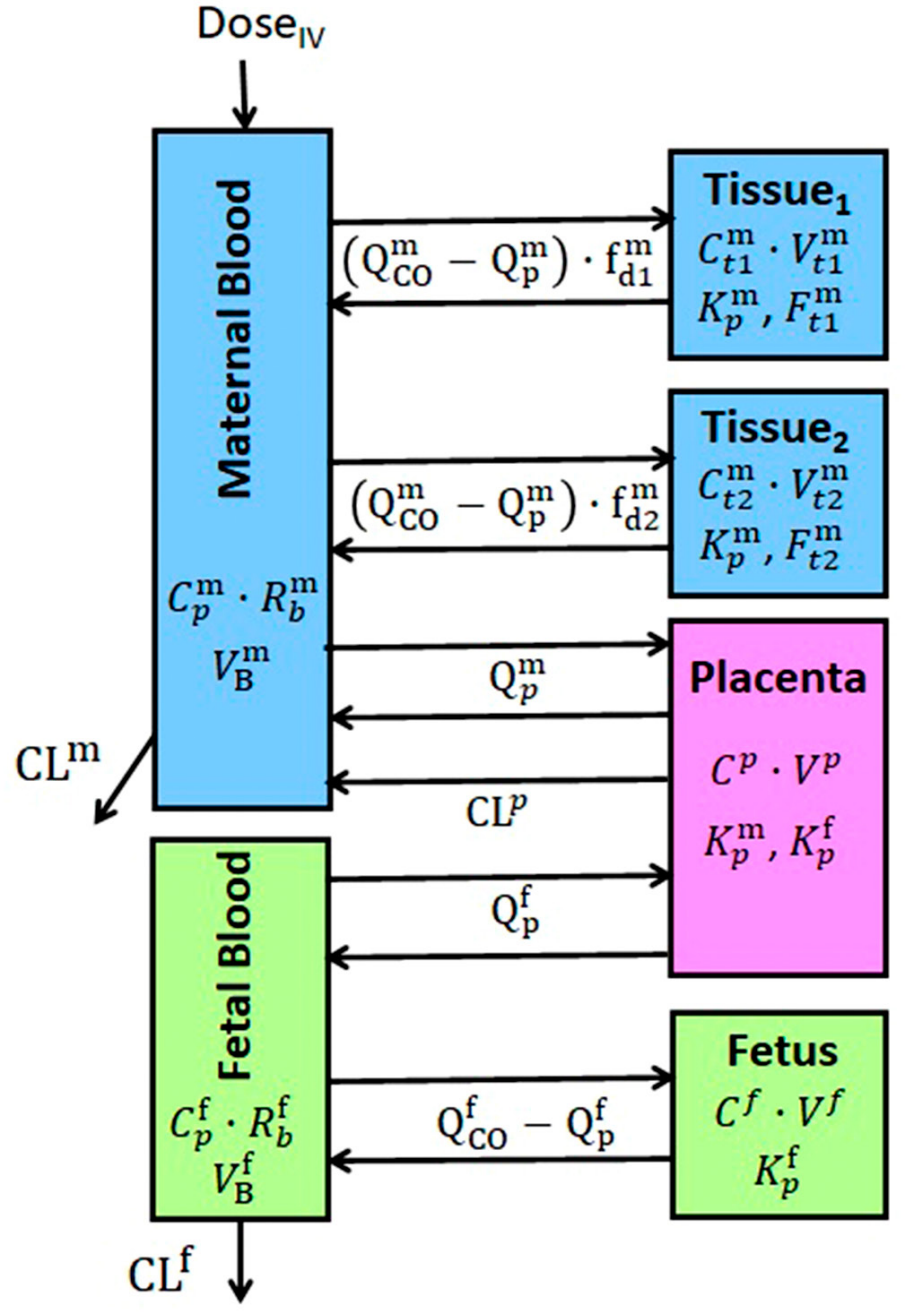
A structure of the minimal physiologically‐based pharmacokinetic model for drugs in pregnant women. Model equations and definitions of model variables and parameters are given in the Methods section and Table 1 and Table S2.

The hybrid mPBPK model consists of a two‐tissue mPBPK model for the mother and a one‐tissue mPBPK model for the fetus that are connected by a model of the placenta. The maternal model was adopted from Song and Jusko^9^ and the fetal/placental model is based on the PBPK model developed by Szeto et al.^13^ The joint model diagram is shown in Figure 1. The model differential equations are:

(1) $$\begin{array}{c} R_{b}^{m}∙\frac{dC_{p}^{m}}{\mathrm{dt}}=\frac{\text{Input}}{V_{B}^{m}}+\left(Q_{\mathrm{CO}}^{m}-Q_{p}^{m}\right)∙f_{d1}^{m}∙\left(\frac{C_{t1}^{m}∙{\;R}_{b}^{m}}{K_{p}^{m}∙V_{B}^{m}}-\frac{C_{p}^{m}∙{\;R}_{b}^{m}}{V_{B}^{m}}\right)\\ \;+{\mathrm{CL}}^{p}∙\frac{C^{p}∙R_{b}^{f}}{V_{B}^{m}}+\left(Q_{\mathrm{CO}}^{m}-Q_{p}^{m}\right)∙f_{d2}^{m}∙\left(\frac{C_{t2}^{m}∙{\;R}_{b}^{m}}{K_{p}^{m}∙V_{B}^{m}}-\frac{C_{p}^{m}∙{\;R}_{b}^{m}}{V_{B}^{m}}\right)\\ {\;+Q}_{p}^{m}∙\left(\frac{C^{p}∙{\;R}_{b}^{m}}{K_{p}^{m}∙V_{B}^{m}}-\frac{C_{p}^{m}∙{\;R}_{b}^{m}}{V_{B}^{m}}\right)-{\mathrm{CL}}^{m}∙\;\frac{C_{p}^{m}∙R_{b}^{m}}{V_{B}^{m}} \end{array}$$

(2) $$\begin{array}{c} \frac{dC_{t1}^{m}}{\mathrm{dt}}=\left(Q_{\mathrm{CO}}^{m}-Q_{p}^{m}\right)∙f_{d1}^{m}∙\left(\frac{C_{p}^{m}∙R_{b}^{m}}{V_{t1}^{m}}-\frac{C_{t1}^{m}∙{\;R}_{b}^{m}}{K_{p}^{m}∙V_{t1}^{m}}\right) \end{array}$$

(3) $$\begin{array}{c} \frac{dC_{t2}^{m}}{\mathrm{dt}}=\left(Q_{\mathrm{CO}}^{m}-Q_{p}^{m}\right)∙f_{d2}^{m}∙\left(\frac{C_{p}^{m}∙{\;R}_{b}^{m}}{V_{t2}^{m}}-\frac{C_{t2}^{m}∙{\;R}_{b}^{m}}{K_{p}^{m}∙V_{t2}^{m}}\right) \end{array}$$

(4) $$\begin{array}{c} \frac{dC^{p}}{\mathrm{dt}}=Q_{p}^{m}∙\left(\frac{C_{p}^{m}∙{\;R}_{b}^{m}}{V^{p}}-\frac{C^{p}∙{\;R}_{b}^{m}}{K_{p}^{m}∙V^{p}}\right)\\ \;+Q_{p}^{f}∙\left(\frac{C_{p}^{f}∙R_{b}^{f}}{V^{p}}-\frac{C^{p}∙{\;R}_{b}^{f}}{K_{p}^{f}V^{p}}\right)-{\mathrm{CL}}^{p}∙\frac{C^{p}∙{\;R}_{b}^{m}}{V^{p}} \end{array}$$

(5) $$\begin{array}{c} R_{b}^{f}∙\frac{dC_{p}^{f}}{\mathrm{dt}}=\left(Q_{\mathrm{CO}}^{f}-Q_{p}^{f}\right)∙\left(\frac{C^{f}∙{\;R}_{b}^{f}}{K_{p}^{f}∙V_{B}^{f}}-\frac{C_{p}^{f}∙{\;R}_{b}^{f}}{V_{B}^{f}}\right)\\ \;{+Q}_{p}^{f}∙\left(\frac{C^{p}∙R_{b}^{f}}{K_{p}^{f}∙V_{B}^{f}}-\frac{C_{p}^{f}∙{\;R}_{b}^{f}}{V_{B}^{f}}\right)-{\mathrm{CL}}^{f}∙\;\frac{C_{p}^{f}∙R_{b}^{f}}{V_{B}^{f}} \end{array}$$

(6) $$\begin{array}{c} \frac{dC^{f}}{\mathrm{dt}}=\left(Q_{\mathrm{CO}}^{f}-Q_{p}^{f}\right)∙\left(\frac{C_{p}^{f}∙{\;R}_{b}^{f}}{V^{f}}-\frac{C^{f}∙{\;R}_{b}^{f}}{K_{p}^{f}∙V^{f}}\right) \end{array}$$

with all zero initial conditions indicating no drug in the system before the first dose at time 0:

(7) $$C_{p}^{m}\left(0\right)=0,C_{t1}^{m}\left(0\right)=0,C_{t2}^{m}\left(0\right)=0,C^{p}\left(0\right)=0,C_{p}^{f}\left(0\right)=0,C^{f}\left(0\right)=0$$

The Input represents the drug input rate into the maternal plasma such as an intravenous (i.v.) bolus dose, infusion, or first‐order rate. The superscripts refer to maternal (^m^), fetal (^f^), and placental (^p^) variables or parameters. $C_{p}^{m}$, $C_{t1}^{m}$, and $C_{t2}^{m}$ are drug concentrations in maternal plasma, Tissue_1_, and Tissue_2_; $C_{p}^{f}$ and $C^{f}$ are drug concentrations in fetal plasma and fetus (considered as a fetal tissue), and $C^{p}$ denotes drug concentration in the placenta. $\;R_{b}^{m}$ and $R_{b}^{f}$ are the maternal and fetal blood‐to‐plasma ratios, so the products $R_{b}^{m}{∙C}_{p}^{m}$ and $R_{b}^{f}∙C_{p}^{f}$ are equal to the drug concentrations in maternal and fetal blood. $K_{p}^{m}$ and $K_{p}^{f}$ represent maternal and fetal tissue‐to‐plasma partition coefficients. The maternal blood flow in the placenta $Q_{p}^{m}$ is subtracted from the maternal cardiac output $Q_{\mathrm{CO}}^{m}$ to account for the blood flow through Tissue_1_ and Tissue_2_ as determined by the fractions $f_{d1}^{m}$ and $f_{d2}^{m}$. We assume $f_{d1}^{m}+f_{d2}^{m}=1$. Analogously, the fetal blood flow through the placenta $Q_{p}^{f}$ is subtracted from the fetal cardiac output $Q_{\mathrm{CO}}^{f}$ to account for the blood flow through the fetus. ${\mathrm{CL}}^{m}$ and ${\mathrm{CL}}^{f}$ denote maternal and fetal elimination clearances of drug from the blood, whereas CL^p^ reflects placental efflux clearance. The right‐hand sides of the model differential equations are divided by the corresponding blood/tissue volumes $V_{B}^{m}$, $V_{t1}^{m}$, $V_{t2}^{m}$, $V^{p}$, $V_{B}^{f}$, and $V^{f}$ to express model‐dependent variables as concentrations rather than amounts.

### Data

We searched the literature for data on DEX and BET in parturient women and attempted to obtain raw data from all authors of published (and known unpublished) studies. The data consisted of time courses of maternal plasma concentrations of these CSs (*n* = 71 for DEX and 333 for BET) and single measurements of fetal plasma concentrations based on blood samples from the umbilical cord vein at birth (*n* = 14 for DEX and 232 for BET). Any data that were missing information on the dosing times or CS formulation were not used. The data were digitized and converted to a numerical format available for PK analysis. The naïve pooled data analysis approach was applied in the absence of individual information. The sources of data, drug formulation, route of administration, and dosing regimens are listed in Table S1.

### mPBPK model for antenatal CSs

The hybrid mPBPK model defined by Equations ((1), (2), (3), (4), (5), (6), (7)) was applied to describe the PK of DEX and BET in parturient women. The Input consisted of either the i.v. bolus doses, oral (p.o.) administration ($A_{\mathrm{PO}})$, or intramuscular (i.m.) injections ($A_{\mathrm{IM}})$:

(8) $$\begin{array}{c} \text{Input}=\sum_{i=1}^{n_{\mathrm{IV}}}{\text{Dose}}_{\mathrm{IV}}∙\delta \left(t-t_{i}\right)+k_{\mathrm{aPO}}∙A_{\mathrm{PO}}\\ \;+k_{\mathrm{aIM}}∙A_{\mathrm{IM}}+k_{\text{aIMa}}∙A_{\mathrm{IMa}} \end{array}$$

where $k_{\mathrm{aPO}}$, $k_{\mathrm{aIM}}$, $k_{\text{aIMa}}$ are the first‐order rate constants and $0=t_{1}$,…, $t_{\mathrm{nIV}}$ are the dosing times. The term ${\text{Dose}}_{\mathrm{IV}}∙\delta \left(t-t_{i}\right)$ denotes a bolus ${\text{Dose}}_{\mathrm{IV}}$ administered at time $t_{i}$. Because BET was also an i.m. administration as a 1:1 mixture of phosphate and acetate (BET‐PA), $A_{\mathrm{IMa}}$ describes the amount of BET in the i.m. depot originating from the acetate portion of the dose. The differential equations for the first‐order input processes are:

(9) $$\frac{dA_{\mathrm{PO}}}{\mathrm{dt}}=\sum_{i=1}^{n_{PO}}F_{\mathrm{PO}}∙{\text{Dose}}_{\mathrm{PO}}∙\delta \left(t-t_{i}\right)-k_{\mathrm{aPO}}∙A_{\mathrm{PO}}$$

(10) $$\begin{array}{c} \frac{dA_{\mathrm{IM}}}{\mathrm{dt}}=\sum_{i=1}^{n_{\mathrm{IM}}}F_{\mathrm{IM}}∙{\text{Dose}}_{\mathrm{IM}}∙\delta \left(t-t_{i}\right)-k_{\mathrm{aIM}}∙A_{\mathrm{IM}} \end{array}$$

(11) $$\begin{array}{c} \frac{dA_{\mathrm{IMa}}}{\mathrm{dt}}=\sum_{i=1}^{n_{\mathrm{IM}}}F_{\mathrm{IMa}}∙{\text{Dose}}_{\mathrm{IMa}}∙\delta \left(t-t_{i}\right)-k_{\text{aIMa}}∙A_{\mathrm{IMa}} \end{array}$$

where $F_{\mathrm{PO}}$, $F_{\mathrm{IM}}$, and $F_{\mathrm{IMa}}$ denote the bioavailabilities. In the case of i.m. BET‐PA administration: ${\text{Dose}}_{\mathrm{IM}}={\text{Dose}}_{\mathrm{IMa}}=0.5∙\text{Dose}$. Because of the bolus inputs in Equations ((9), (10), (11)), zero initial conditions were assumed:

(12) $$\begin{array}{c} A_{\mathrm{PO}}\left(0\right)=0,A_{\mathrm{IM}}\left(0\right)=0,A_{\mathrm{IMa}}\left(0\right)=0\; \end{array}$$

A diagram of the hybrid mPBPK model for BET and DEX that includes the input processes is shown in Figure S1.

### Model calibration

Most model parameters were obtained from the literature. The volume of maternal tissue was calculated based on the typical body weight of pregnant women (${\mathrm{BW}}^{m}$) at 39 weeks of gestation corrected by the volumes of maternal blood ($V_{B}^{m})$, fetus ($V^{f})$, amniotic fluid ($V_{a})$, and placenta ($V^{p}$):

(13) $$V_{t}^{m}={\mathrm{BW}}^{m}/\rho \;-\;V_{B}^{m}-\;V^{f}-V^{p}-V_{a}$$

where the tissue density ρ was assumed to be 1 kg/L.^14^ $V_{t}^{m}$ was used for the volumes of Tissue_1_ and Tissue_2_:

(14) $$V_{t1}^{m}=F_{t1}^{m}∙{\;V}_{t}^{m}\;\text{and}\;V_{t2}^{m}=F_{t2}^{m}\;∙{\;V}_{t}^{m}$$

where the fractions add up to 1: $F_{t2}^{m}+F_{t1}^{m}=1$. The volume of fetal tissue $V_{t}^{f}$ was obtained from $V^{f}$ upon correction for the fetoplacental blood volume $V_{B}^{f}$:

(15) $$V_{t}^{f}=V^{f}-V_{B}^{f}∙V^{f}/\left(V^{f}+V^{p}\right)$$

The fetal clearance CL^f^ was calculated as a fraction of CL^m^ based on a 33.8‐fold greater intrinsic clearance of 7‐hydroxy‐dehydroepiandrosterone by cytochrome P450 (CYP) 3A4 in the mother versus CYP3A7 expression in the fetus.^15^ The blood‐to‐plasma ratios $R_{b}^{m}\;$ and $R_{b}^{f}$ were set to 1. The remaining model parameters were estimated by the simultaneous fitting of the mPBPK models for DEX and BET to the maternal and fetal plasma concentration time courses obtained from the literature. The data and model predictions of $C_{\text{pDEX}}^{m}$, $C_{\text{pDEX}}^{f}$, $C_{\text{pBET}}^{m}$, and $C_{\text{pBET}}^{f}$ were log‐transformed. The additive residual error model was applied with different errors for DEX and BET maternal and fetal observations. The first‐order conditional estimation method implemented in NONMEM 7.4 (ICON plc, Dublin, Ireland) was applied. The values and sources of the physiological parameters are listed in Table S2.

### Simulations

The hybrid mPBPK model and fitted parameters were used to simulate fetal‐to‐maternal plasma concentration ratios $C_{p}^{f}/C_{p}^{m}$ for DEX‐P and BET‐P given as single 8‐mg i.v. and i.m. doses. Because the mPBPK model is linear with dose, the simulated curves represent all single doses.

The hybrid mPBPK model was also used to simulate time courses of maternal and fetal BET and DEX plasma concentrations for several clinically relevant dosing regimens. The plots included the average and 5th and 95th prediction intervals. The latter were calculated as the 5th and 95th percentiles of log‐normally distributed residual errors around the model‐predicted value. The area under the curve (AUC) calculation for simulated concentrations was performed using the trapezoidal rule. The terminal half‐life was calculated from the slope of the simulated curves at *t* = 200 h. Simulations were performed using NONMEM 7.4, and the plots were generated using RStudio 1.4 (RStudio, PBC).

### Sensitivity analysis

To determine the impact of key model parameters on the simulated values of maternal maximum concentration $\left(C_{\max}^{m}\right)$, $\text{fetal maximum concentration}\;\left(C_{\max}^{f}\right)$, $\text{maternal trough concentration}\;(C_{\text{trough})}^{m}$, fetal trough concentration $\left(C_{\text{trough}}^{f}\right)$, maternal AUC for the time interval 0–72 h $\left({\mathrm{AUC}}_{0–72}^{m}\right)$, fetal AUC for the time interval 0–72 h $\left({\mathrm{AUC}}_{0–72}^{f}\right)$, drug concentrations in maternal plasma $\left(C_{p}^{m}\right)$, and drug concentrations in fetal plasma ($C_{p}^{f}),$ we performed a global sensitivity analysis (GSA). The parameters assessed were ${\mathrm{CL}}^{m}$, ${\mathrm{CL}}^{f}$, ${\mathrm{CL}}^{p}$, $K_{p}^{m}$, $K_{p}^{f}$, $V_{B}^{m}$, $V_{B}^{f}$, $V_{t}^{m}$, $V_{t}^{f}$, $V^{p}$, $Q_{\mathrm{CO}}^{m}$, $Q_{\mathrm{CO}}^{f}$, $Q_{p}^{m}$, $Q_{p}^{f}$, and $k_{\mathrm{aIM}}$. The Sobol first‐order and total‐order indices were calculated as described by Sala et al.^16^ The GSA was performed in the MATLAB R2020b toolbox SimBiology 6.0 (Mathworks). The Sobol sampling method was applied with the 40,000 samples given a 15‐parameter space.

## RESULTS

### Parameter estimation and fitting data

**FIGURE 2:**
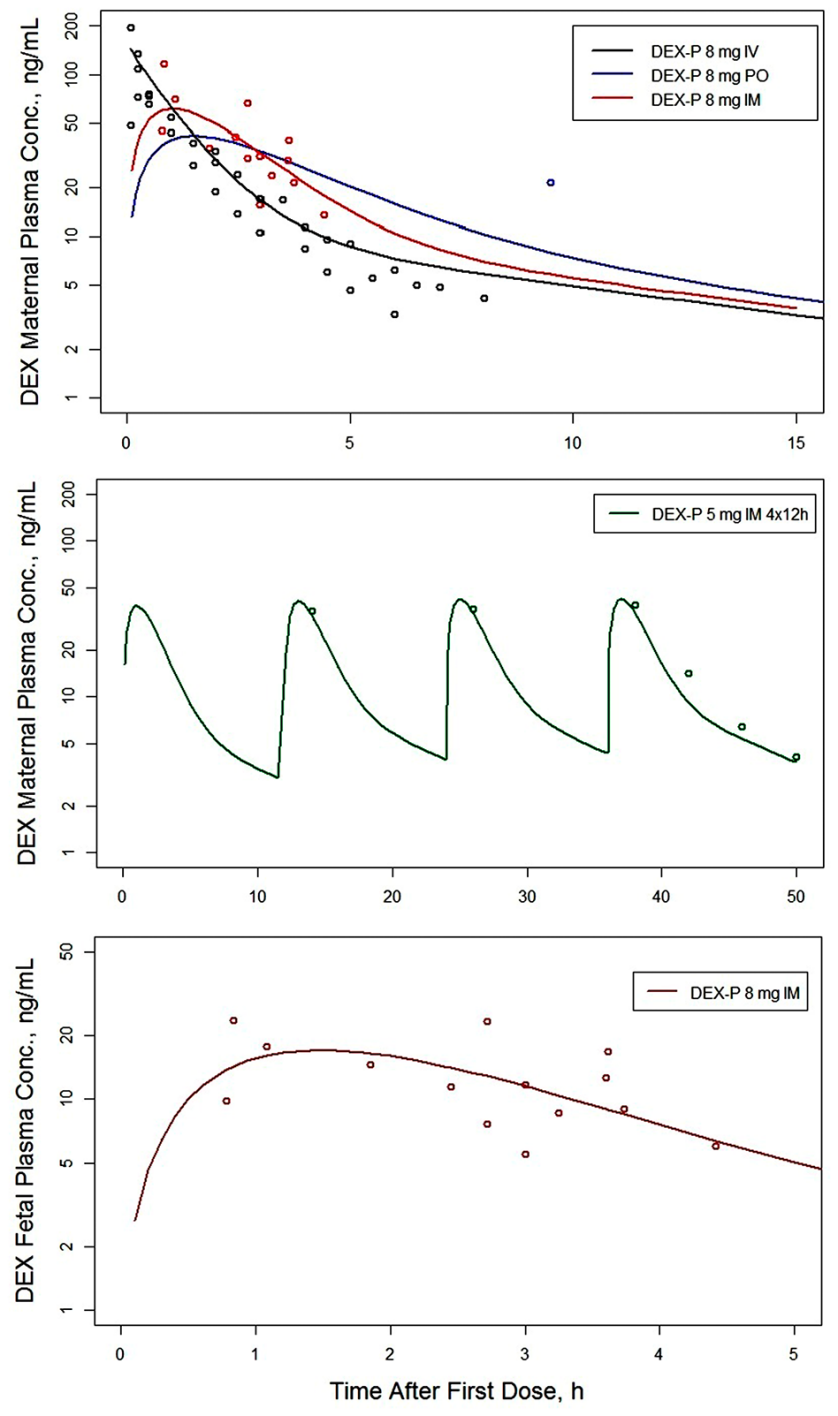
Observed maternal and fetal plasma concentrations (symbols) of DEX overlaid with minimal physiologically‐based pharmacokinetic model‐fitted curves following DEX‐P administration. The legends inform about the route of administration, dose, and dosing intervals. DEX, dexamethasone; DEX‐P, dexamethasone phosphate; IM, intramuscular; IV, intravenous; PO, oral.

**FIGURE 3:**
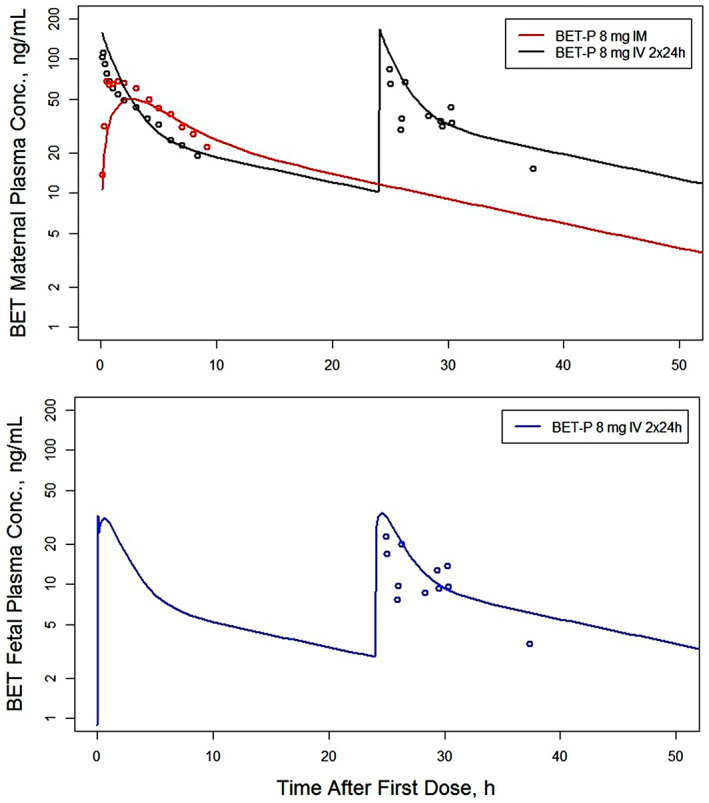
Observed maternal and fetal plasma concentrations (symbols) of BET overlaid with minimal physiologically‐based pharmacokinetic model‐fitted curves following BET‐P administration. The legends inform about the route of administration, dose, and dosing intervals. BET, betamethasone; BET‐P, betamethasone phosphate; IM, intramuscular; IV, intravenous.

**FIGURE 4:**
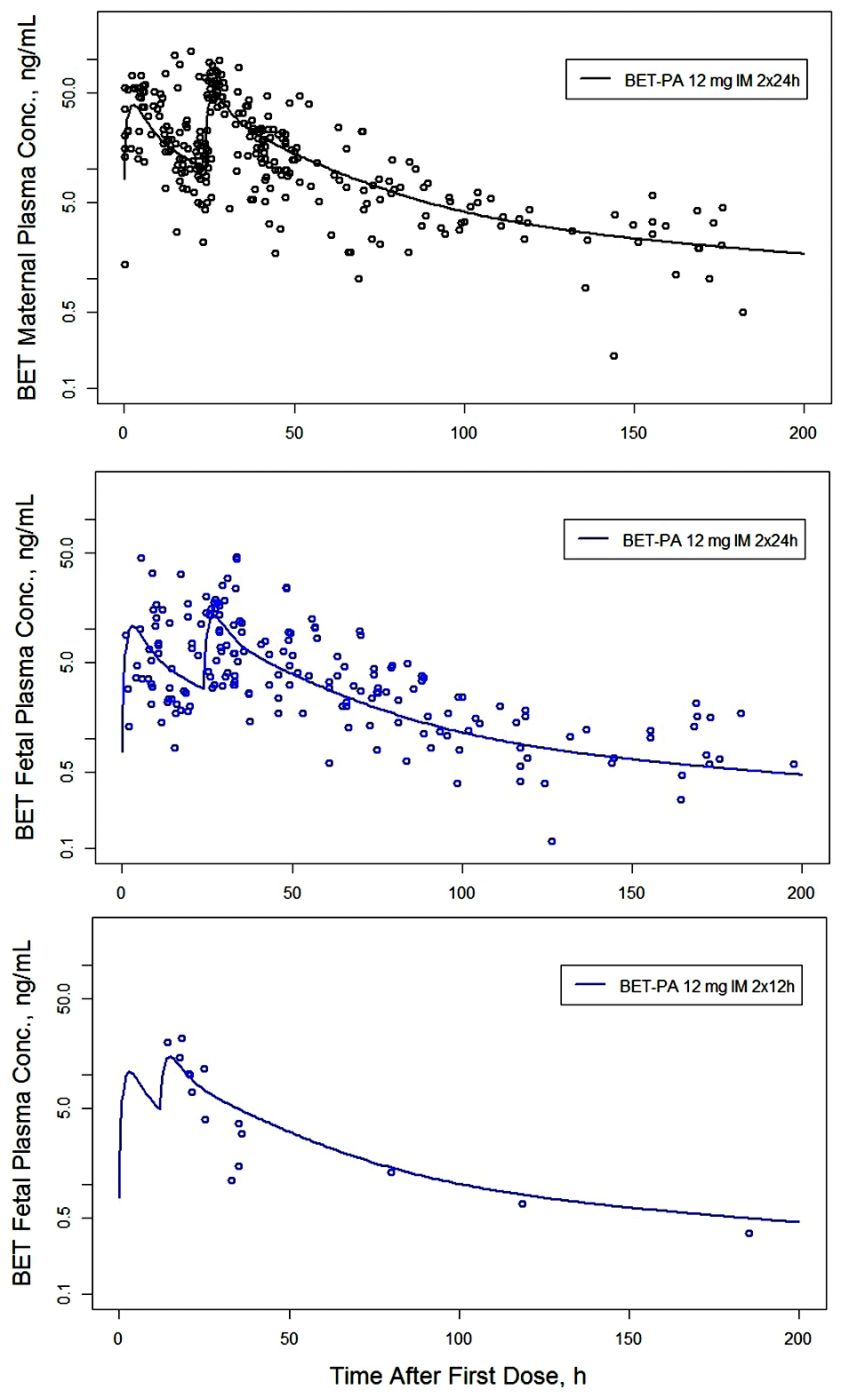
Observed maternal and fetal plasma concentrations (symbols) of BET overlaid with minimal physiologically‐based pharmacokinetic model‐fitted curves following BET‐PA administration. The legends inform about the route of administration, dose, and dosing intervals. BET, betamethasone; BET‐PA, betamethasone phosphate and acetate; IM, intramuscular.

The drug‐specific model parameters were estimated by a simultaneous fitting of all suitable DEX and BET data with the sources summarized in Table S1. The fitted curves overlaid with the data are shown in Figure 2 for maternal and fetal plasma concentrations of DEX and correspondingly in Figure 3 for BET and Figure 4 for BET‐PA. Overall, the fitted curves well captured the relatively rich and diverse maternal plasma concentrations and less dense fetal plasma concentrations. The exceptions are fittings of maternal plasma concentrations for DEX following 6 mg i.m. and 8 mg p.o., where there was modest underprediction. The literature data for BET‐PA 12 mg 2 × 24 h provided the most extensive maternal and fetal concentrations spanning 200 h because this is the primary treatment method of pregnant women with antenatal CSs in the United States and England.

The hybrid mPBPK parameter estimates are presented in Table 1. The maternal disposition clearance of DEX was three times that of BET. The relative standard errors of parameter estimates are below 42%. The absolute values of correlation coefficients between parameter estimates were <0.7 with the exception of the correlation between $K_{p}^{m}\;$ and ${\mathrm{CL}}_{\mathrm{DEX}}^{p}$ and $k_{\text{aIMa}}$ and $F_{\mathrm{IMa}},$ where it was close to 0.9. The estimates of bioavailabilities for i.m. and p.o. doses were at the upper limit of 1 and, ultimately, they were fixed at this value. The estimates of the variances of the residual error for log‐transformed data were $\sigma_{\mathrm{DEX}}^{2m}=0.185,\sigma_{\mathrm{DEX}}^{2f}=0.157,\sigma_{\mathrm{BET}}^{2m}=0.510,\text{and}\;\sigma_{\mathrm{BET}}^{2f}=0.691.$ These imply about 40% coefficient of variation (CV) in the residual variability of the DEX data and 70%–80% CV for the BET data. Such sizable variability is expected for the multisource data obtained from the literature.

**TABLE 1:** The minimal physiologically‐based pharmacokinetic model parameter estimates and their %RSE obtained from simultaneous fitting of DEX and BET data

Parameter	Definition	Estimate (%RSE)
${\mathrm{CL}}_{\mathrm{DEX}}^{m}$, L/h	DEX maternal blood elimination clearance	26.7 (13.1)
${\mathrm{CL}}_{\mathrm{BET}}^{m}$, L/h	BET maternal blood elimination clearance	8.93 (7.6)
${\mathrm{CL}}_{\mathrm{DEX}}^{p}$, L/h	DEX placental blood clearance^a^	36.1 (30.5)
${\mathrm{CL}}_{\mathrm{BET}}^{p}$, L/h	BET placental blood clearance^a^	42.0 (35.7)
$K_{p}^{m}$	Maternal tissue‐to‐plasma partition coefficient	2.37 (27.5)
$f_{d1}^{m}$	Fraction of cardiac output for tissue_1_	0.953 (1.0)
$F_{t1}^{m}$	Fraction of total tissue volume for tissue_1_	0.234 (41.2)
k_aPODEX_, h^−1^	Absorption rate from GI tract	0.446 (28.7)
$F_{\mathrm{PO}}$	Oral bioavailability	1^b^
$k_{\text{aIMDEX}}$, h^−1^	Absorption rate from muscle for DEX	0.893 (29.5)
$k_{\text{aIMBET}}$, h^−1^	Absorption rate from muscle for BET	0.335 (24.3)
$F_{\mathrm{IM}}$	Intramuscular bioavailability	1^b^
$k_{\text{aIMa}}$, h^−1^	Absorption rate from muscle for BET acetate	0.00534 (13.5)
$F_{\mathrm{IMa}}$	Intra‐muscular bioavailability for BET acetate	0.575 (27.7)
$\sigma_{\mathrm{DEX}}^{2m}$	Variance of residual error for maternal DEX observations	0.185 (24.8)
$\sigma_{\mathrm{DEX}}^{2f}$	Variance of residual error for fetal DEX observations	0.157 (1.0)
$\sigma_{\mathrm{BET}}^{2m}$	Variance of residual error for maternal BET observations	0.510 (8.4)
$\sigma_{\mathrm{BET}}^{2f}$	Variance of residual error for fetal BET observations	0.691 (12.0)

Abbreviations: BET, betamethasone; DEX, dexamethasone; GI, gastrointestinal; RSE, relative standard error.

^a^ As affected by net permeability and *P*‐*glycoprotein* efflux.

^b^ Parameter was fixed.

Of importance, the major determinants of fetal exposures and fetal/maternal concentration ratios, besides the material plasma concentrations, are the placental blood flow and the placental clearance (CL^p^) with the latter reflecting *P*‐*glycoprotein* (P‐gp)–mediated efflux. The former were literature values applied to both drugs, whereas the model‐fitting yielded mean CL^p^ values of 42.0 for BET and 36.1 L/h for DEX. Considering the sources and variability in the data, these CL^p^ values are essentially the same. Indeed, when one value was applied, the joint CL^p^ was 40.4 L/h (37.6%) with imperceptible effect on fitting of the data.

Following i.v. bolus injections of 8‐mg DEX‐P or BET‐P at time 0 h, the model for both DEX and BET predicted fetal plasma concentrations with immediate peaks of $C_{\text{maxDEX}}^{f}=24.7$ ng/ml and $C_{\text{maxBET}}^{f}=32.5$ ng/ml followed by secondary peaks of $C_{\text{maxDEX}}^{f}=28.0$ ng/ml and $C_{\text{maxBET}}^{f}=30.9\;$ ng/ml that occurred at $t_{\text{maxDEX}}^{f}=0.4$ h and $t_{\text{maxBET}}^{f}=0.6$ h (see Figures 2 and 3 peak times (t_max_)). The peaks for model‐predicted maternal plasma concentrations occurred at time 0 h and were $C_{\text{maxDEX}}^{m}=145.2$ ng/ml and $C_{\text{maxBET}}^{m}=157.3$ ng/ml. The half‐lives calculated from the terminal slopes of predicted curves were $t_{1/2\mathrm{DEX}}^{m}=8.3$ h and $t_{1/2\mathrm{BET}}^{m}=16.5$ h for BET‐P. The half‐lives for fetal plasma concentrations were very similar to the maternal plasma concentrations. The i.m. injection of 8‐mg DEX‐P resulted in maternal and fetal peaks of $C_{\text{maxDEX}}^{m}=61.3\;$ ng/ml and $C_{\text{maxDEX}}^{f}=17.0$ ng/ml that occurred at $t_{\text{maxDEX}}^{m}=0.9$ h and $t_{\text{maxDEX}}^{f}=1.6$ h (see Figure 2). The analogous values following the i.m. injection of 8‐mg BET‐P were $C_{\text{maxBET}}^{m}=50.3$ ng/ml and $C_{\text{maxBET}}^{f}=13.9$ ng/ml and $t_{\text{maxBET}}^{m}=3$ h and $t_{\text{maxBET}}^{f}=4$ h (see Figure 3). The fitted curve to the BET data following the two i.m. injections of 12 mg of BET‐PA exhibited peaks for maternal and fetal plasma concentrations of $C_{\text{maxBET}}^{m}=38.3$ ng/ml and $C_{\text{maxBET}}^{f}=10.6\;$ ng/ml and $t_{\text{maxBET}}^{m}=3$ h and $t_{\text{maxBET}}^{f}=3$ h (see Figure 4). The terminal half‐life from BET‐PA (117 h) was prolonged due to the slow hydrolysis and release from the injection site. The PK data for the p.o. doses were available only for DEX‐P at 8 mg. The model‐predicted peaks were $C_{\text{maxDEX}}^{m}=41.8$ ng/ml and $C_{\text{maxDEX}}^{f}=12.1$ ng/ml that occurred at $t_{\text{maxDEX}}^{m}=1.6$ h and $t_{\text{maxDEX}}^{f}=2.0$ h (see Figure 2).

### Simulations

To assess the time necessary to reach an equilibrium between fetal and maternal plasma concentrations of the CSs, we used the mPBPK model to simulate the ratios of $C_{p}^{f}/C_{p}^{m}$ for DEX‐P and BET‐P given as single i.v. and i.m. doses (see Figure S2). The $C_{p}^{f}/C_{p}^{m}$ curves exhibited a peak before they reached a constant ratio. For DEX‐P, the peak ratios of 0.40 (i.v.) and 0.33 (i.m.) occurred at 2.4 h and 5 h. The plateau ratio of 0.311 was reached at about 20 h after both i.v. and i.m. doses. The peaks for BET‐P were smaller at 0.32 (i.v.) and 0.29 (i.m.), but they occurred at similar times of 2 h and 6 h. The plateau of 0.282 for BET‐P was reached about the same time as the plateau of 0.311 for DEX‐P.

**FIGURE 5:**
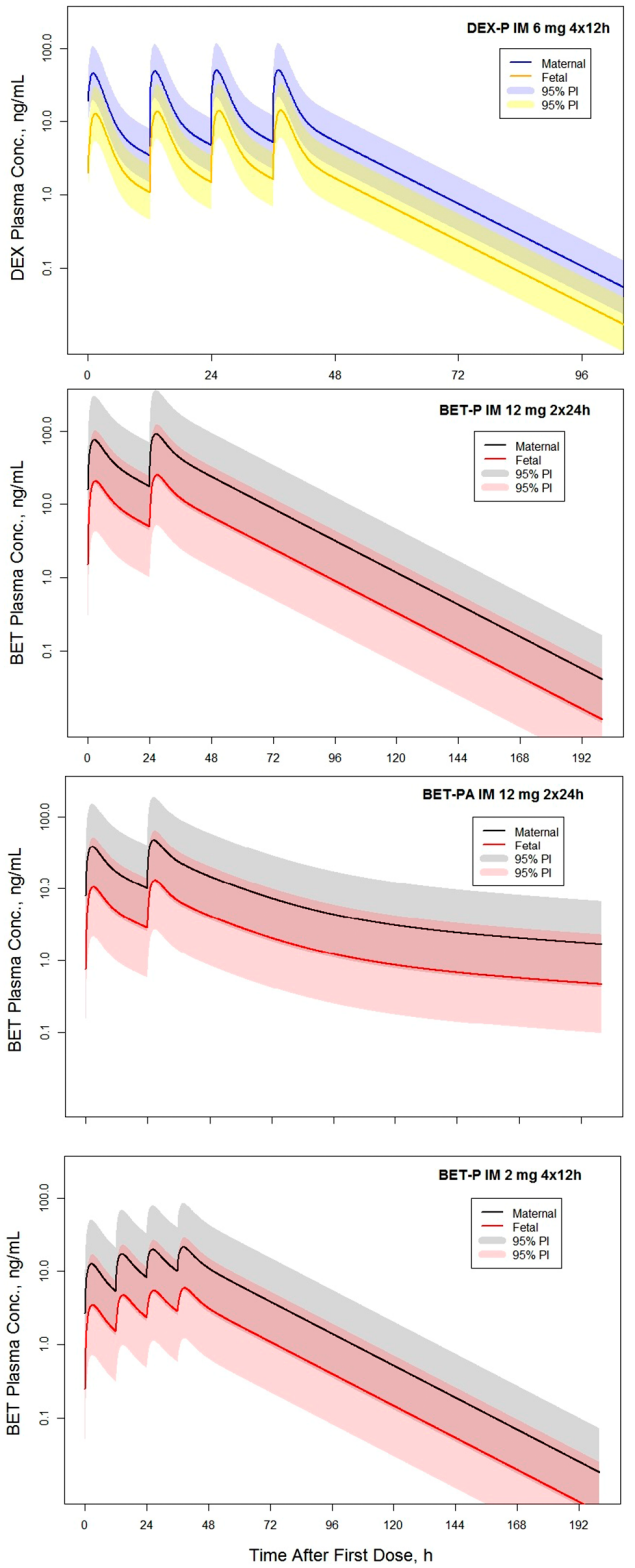
Simulated corticosteroid maternal and fetal plasma concentrations for indicated dosing regimens. The solid lines represent model predictions. The shaded regions represent the 5th and 95th PIs. Parameters used for simulations are listed in Tables 1 and S2. BET, betamethasone; BET‐P, betamethasone phosphate; DEX, dexamethasone; DEX‐P, dexamethasone phosphate; IM, intramuscular; PI, prediction interval.

Simulations of the four regimens for the antenatal CSs recommended by WHO were performed to assess the expected maternal and fetal exposures to BET and DEX.^12^ The tested regimens included four i.m. doses of 6‐mg DEX‐P every 12 h (DEX‐P i.m. 6 mg 4 × 12 h), two i.m. doses of 12‐mg BET‐P every 24 h (BET‐P i.m. 12 mg 2 x 24 h), two i.m. doses of 12‐mg BET‐PA every 24 h (BET‐PA i.m. 12 mg 2 x 24 h) and BET‐P i.m. (2 mg 4 x 12 h) (see Figure 5). The values of maximum concentration, trough concentration, and AUC for the time interval 0–72 h are shown in Table 2. The terminal half‐lives for the mother and fetus were identical. These half‐life values were 8.3 h (DEX‐P), 16.5 h (BET‐P), and 117 h (BET‐PA). These values were consistent with the half‐lives calculated for single doses. The $C_{\max}^{m}$ and $C_{\max}^{f}$ values were similar for DEX‐P and BET‐PA regimens but almost twice higher for BET‐P. The lowest 48‐h troughs $C_{\text{trough}}^{m}$ and $C_{\text{trough}}^{f}$ were for DEX‐P. They almost doubled for BET‐PA and tripled for BET‐P. The $\mathrm{AU}C_{0–72}^{m}$ was lowest for DEX‐P, increased 2.6‐fold for BET‐P, and was 1.5‐fold for BET‐PA. The ratios of $\mathrm{AU}C_{0–72}^{f}/\mathrm{AU}C_{0–72}^{m}$ were approximately 0.28 for all dosing regimens.

**TABLE 2:** Simulated maximum and trough before the last dose maternal and fetal plasma concentrations and area under the curve after the first dose values for the four dosing regimens shown in Figure 5

Regimen	$C_{\max}^{m}\;$ ng/ml	$C_{\max}^{f}$ ng/ml	$C_{\text{trough}}^{m}\;$ ng/ml	$C_{\text{trough}}^{f}$ ng/ml	$\mathrm{AU}C_{0–72}^{m}\;$ ng/ml ⋅ h	$\mathrm{AU}C_{0–72}^{f}$ ng/ml ⋅ h
DEX‐P i.m. 6 mg 4 × 12 h	50.8	14.2	5.2	1.6	879	266
BET‐P i.m. 12 mg 2 × 24 h	92.0	25.3	17.6	5.0	2390	676
BET‐PA i.m. 12 mg 2 × 24 h	48.0	13.2	10.1	2.9	1368	378
BET‐P i.m. 2 mg 4 × 12 h	21.5	5.9	10.0	2.8	796	221

Abbreviations: $\mathrm{AU}C_{0–72}^{f},$ fetal area under the curve for the time interval 0–72 h; $\mathrm{AU}C_{0–72}^{m},$ maternal area under the curve for the time interval 0–72 h; BET‐P, betamethasone phosphate; BET‐PA, betamethasone phosphate and acetate; $C_{\max}^{f},$ fetal maximum concentration; $C_{\max}^{m},$ maternal maximum concentration; $C_{\text{trough}}^{f},$ fetal trough concentration; $C_{\text{trough}}^{m},$ maternal trough concentration; DEX‐P, dexamethasone phosphate; i.m., intramuscular.

### Sensitivity analysis

We applied GSA to determine the impact of the 15 selected model parameters on each of the following outputs: $C_{\max}^{m}$, $C_{\max}^{f}$, $C_{\text{trough}}^{m}$, $C_{\text{trough}}^{f}$, ${\mathrm{AUC}}_{0–72}^{m}$, ${\mathrm{AUC}}_{0–72}^{f}$, $C_{p}^{m}$, and $C_{p}^{f}$. The regimens DEX‐P i.m. 6 mg 4 × 12 h and BET‐P i.m. 12 mg 2 × 24 h were tested (see Table 2). For all outputs and all parameters, the Sobol first‐order and total‐order indices indicating the fractional contributions to the overall variability were very similar. For DEX, the parameters contributing most to the variability of $C_{\max}^{m}$ and $C_{\text{trough}}^{m}\;$were ${\mathrm{CL}}^{m}$, $k_{\mathrm{aIM}}$, $K_{p}^{m}$, and $V_{t}^{m}$ (Sobol first‐order index range, 0.17–0.37), whereas $C_{\max}^{f}$ and $C_{\text{trough}}^{f}$were most affected by $K_{p}^{f}$, $Q_{p}^{m}$, ${\mathrm{CL}}^{p}$, ${\mathrm{CL}}^{m}$, and $k_{\mathrm{aIM}}$ (0.07–0.45) (see Figure S3). The ${\mathrm{AUC}}_{0–72}^{m}$ was dominated by only one parameter, ${\mathrm{CL}}^{m}$ (Sobol first‐order index 1), whereas ${\mathrm{AUC}}_{0–72}^{f}$ was controlled by $K_{p}^{f}$, ${\mathrm{CL}}^{m},Q_{p}^{m}$, and ${\mathrm{CL}}^{p}$ (0.16–0.34) (see Figure S3). Parameters contributing most to the variability of the time courses of $C_{p}^{m}$ and $C_{p}^{f}$ coincided with those affecting $C_{\max}^{m}$ and $C_{\max}^{f}$ (see Figure S4). Similar to DEX, for BET, the parameters contributing most to the variability of $C_{\max}^{m}$ were $k_{\mathrm{aIM}},{\mathrm{CL}}^{m}$, $K_{p}^{m}$, and $V_{t}^{m}$ (0.19–0.3), whereas $C_{\max}^{f}$ was most affected by $K_{p}^{f}$, $Q_{p}^{m}$, ${\mathrm{CL}}^{p}$, and ${\mathrm{CL}}^{m}$ (0.19–0.43) (see Figure S5). The variability of $C_{\text{trough}}^{m}$ was determined only by two parameters ${\mathrm{CL}}^{m}$ and $Q_{\mathrm{CO}}^{m}$ (0.88, 0.07), and parameters contributing to $C_{\text{trough}}^{f}$ variability were ${\mathrm{CL}}^{m}$, $K_{p}^{f}$, $Q_{p}^{m}$, and ${\mathrm{CL}}^{p}$ (0.13–0.38). The ${\mathrm{AUC}}_{0–72}^{m}$ was controlled by ${\mathrm{CL}}^{m}$ (Sobol first‐order index 0.92) and ${\mathrm{AUC}}_{0–72}^{f}$ by $K_{p}^{f}$, ${\mathrm{CL}}^{m},Q_{p}^{m}$, and ${\mathrm{CL}}^{p}$ (0.18–0.36) (see Figure S5). As for DEX, the time courses of $C_{p}^{m}$ and $C_{p}^{f}$ are controlled by the same parameters affecting $C_{\max}^{m}$ and $C_{\max}^{f}$ (see Figure S5).

## DISCUSSION

The mPBPK models are considered an intermediate approach between complex full PBPK models and relatively simple, but lacking most physiological features, compartmental models. Similar to full PBPK models, mPBPK models retain the capability to describe time courses of drug concentrations in lumped organs and tissues that are difficult to sample for direct measurements. Full PBPK typically apply tissue:plasma partition coefficients that are based on general prediction methods often found in commercial software without the ability to confirm tissue exposures except in animal studies and by human biopsies. On the other hand, mPBPK models have such composite values based on the fitting of PK data for specific drugs. Both approaches rely on capturing plasma concentration versus time profiles that are, in turn, mostly dependent on having appropriate bioavailability and clearance values (viz. AUC = bioavailability × dose/clearance). So far, mPBPK models have been applied that include brain,^17^ interstitial fluid,^18^ renal tubules,^19^ and endosomes.^20^ To our knowledge, our hybrid mPBPK model is the first to quantify drug exposure in the fetus. Its simplified format compared with full PBPK models offers the advantage of easily performing a “top‐down” meta‐analysis where all available data can be considered for comparison, leveraging ample BET data with limited DEX data, and providing a parsimonious global assessment of the properties of the drugs and system.

The mother and fetus are functionally different but connected organisms that coexist during pregnancy. In addition to the fetus, the placenta and amniotic fluids are integral components in the physiology of PBPK models for pregnant women. Because our objective was to quantify an extensive array of plasma drug concentrations in the mother and fetus, we did not consider a single fetoplacental unit^21^ but, rather, considered the fetus as a separate entity interacting with the mother via the placenta. The placental drug concentrations were described based on the total placental drug concentrations from the PBPK model by Szeto et al.^13^ The separation of maternal and fetal placental components was ignored. To reduce model complexity, the amniotic fluid was also not included. However, its volume was considered when calculating the volume of maternal tissues. The fetal mPBPK model consisted only of a single tissue and fetal blood. These limitations were dictated by the minimalistic approach toward model construction. The two‐tissue structure of the maternal mPBPK was adopted from the multispecies mPBPK model for DEX developed by Song and Jusko.^9^ Their Tissue_1_ represented well‐perfused organs and tissues, whereas Tissue_2_ comprised the remainder of the body with a single tissue‐to‐plasma partition coefficient (*K*_p_) value for both. The proportion of tissue volumes as well as the partitioning of blood flow through the tissues result from estimations using drug‐specific data.

Dynamic changes in physiological and biochemical parameters during pregnancy have been explained by PBPK models using ${\mathrm{BW}}^{m}\;$and gestational age (GA). Because these are highly correlated, GA can used to determine ${\mathrm{BW}}^{m}$,^1^ leaving it as a sole covariate describing time variance of PBPK model parameters. We qualified our hybrid mPBPK model using available data in parturient women, therefore we did not provide relationships between model parameters and GA. For model calibration, we assumed GA = 39 weeks, the GA for a full‐term pregnancy. However, the key parameters leading to maternal and fetal clearances reflect the functioning of the subjects/patients at the time of the original studies and are consistent with literature values.^22^ Relationships connecting maternal and fetal physiologic components to GA can be incorporated into our hybrid mPBPK model if needed. They will need considerations of GA‐dependent changes in CYP3A metabolism^4^ and P‐gp transporter activity^23^ to describe maternal and fetal plasma drug concentrations at other times during pregnancy.

DEX has been reported to induce the CYP3A4 enzyme activity in healthy volunteers and human hepatocyte cultures.^24^ The impact of autoinduction of CS‐metabolizing enzymes on its PK in pregnancy is unknown. Possible autoinduction by DEX in our mPBPK model warrants caution in the interpretation of results of simulations, particularly those involving multiple‐dosing regimens as shown in Figure 5.

The model parameters that were estimated by fitting the maternal and fetal plasma concentration data included clearances, partition coefficients, fractions of the cardiac output and tissue volumes, and absorption parameters (see Table S2). Given the somewhat variable multisource data, the precision of estimates of <42% is adequate. Our estimate of ${\mathrm{CL}}_{\mathrm{DEX}}^{m}=26.7$ L/h is close to the value of 33.5 L/h reported for DEX in parturient women.^25^ Our estimate of ${\mathrm{CL}}_{\mathrm{BET}}^{m}=8.93$ L/h is almost twofold smaller than a value of 17.2 L/h determined for BET in parturient women.^26^ The DEX and BET clearances in pregnant women are higher than in nonpregnant women of 9.5 L/h and 6.5 L/h.^27^ Our estimates of the fraction of the maternal volume attributed to Tissue_1_ of 0.234 was close to the estimate of 0.26 reported for DEX across many species.^9^ The estimate of fraction of cardiac output for Tissue_1_ of 0.953 was higher than a previous 0.85 value.^9^ These parameters were used jointly for BET and DEX along with the maternal tissue:plasma K_p_ value as preliminary fittings yielded similar values. These epimeric CSs have nearly identical structures, similar logP values (DEX, 1.68; BET, 1.9), similar fraction unbound in plasma in pregnancy (DEX, 0.32; BET, 0.36), similar blood:plasma ratios (DEX, 0.93; BET, 1.12), slight renal excretion, and similar other properties except for overall body clearances.^4^ Of special note, Giaginis et al.^28^ reported ex vivo human perfused (cell culture media) placental transfer and metabolism clearance ratios using antipyrine as a control of 0.37 for DEX and 0.41 for BET, indicative of very similar high permeability. Qualitatively, these properties also confirm that these moderately lipophilic CSs well distribute through the entire body as found with DEX PBPK in studies in rats^29^ and that, during pregnancy, the volume of distribution increases. Our estimates of the tissue‐to‐plasma *K*_p_ for the mother (2.37) was higher than previously (1.07), implying factors such as less plasma protein binding, stronger tissue binding, differing role of transporters in pregnancy, or an oversimplified model structure.

The presence of i.v. data (albeit limited) permitted us to estimate the bioavailability of DEX‐P and BET‐P following i.m. and p.o. doses. The estimates were at the physiological boundary of 1 and were thus fixed at this value for convergence of the parameter‐estimating algorithm. This F_IM_ close to 1 agrees with the bioavailability of BET‐P in parturient women who received 8‐mg i.v. and i.m. in two separate studies yielding AUC_IV_ = 31.2 μg/ml ⋅ min^23^ and AUC_IM_ = 29.0 μg/ml ⋅ min.^30^ The estimate of i.m. bioavailability for BET‐acetate was $F_{\mathrm{IMa}}$ = 0.575. The i.m. absorption rate constants were 0.893 h^−1^ (t_1/2_ = 0.78 h) for DEX‐P and 0.335 h^−1^ (*t*_1/2_ = 2.1 h) for BET‐P. The i.m. absorption rate constants in nonpregnant women reported by Krzyzanski et al.^31^ were 0.460 h^−1^ for DEX‐P and 0.971 h^−1^ for BET‐P. The faster i.m. absorption in pregnant women is supported by our data where the observed *t*_max_ values following 8 mg i.m. were 0.25 h^25^ for DEX‐P and 1.5 h^31^ for BET‐P (Figure 2). The estimate of the absorption rate constant following a p.o. dose of DEX‐P was 0.446 h^−1^ and is smaller than an analogous value reported for nonpregnant women of 0.936 h^−1^.^28^ The observed t_max_ value following 8‐mg p.o. DEX was 2 h (Figure 2).^31^ Although differences between the estimates of absorption rates for pregnant and nonpregnant women may be explained by variability in the data, another reason is that estimates of absorption rates will differ when the disposition model changes (unpublished observations). A compartmental model was used previously.^31^

Our simulations show that the equilibrium between fetal and maternal plasma concentrations is reached approximately 20 h after a dose of CS. The ratio $C_{p}^{f}/C_{p}^{m}$ ranging 0.3 to 0.4 is similar for DEX and BET. Tsuei et al.^25^ reported a ratio value of 0.45 with the equilibrium reached at about 5 h after the last dose. No peaks preceding the plateau of the $C_{p}^{f}/C_{p}^{m}$ versus time curve were observed. Petersen et al.^32^ reported $C_{p}^{f}/C_{p}^{m}=0.28$ for BET with no time dependence and the first observation recorded at 1 h after the last dose. Similar findings for BET with $C_{p}^{f}/C_{p}^{m}=0.37\;$were reported by Ballard et al.^33^ A recent study by Zafran et al.^34^ in women dosed with BET‐PA reports the value of $C_{p}^{f}/C_{p}^{m}=0.72$ for BET calculated as the ratio of mean values. The twofold difference from previous values might be explained by a relatively large between‐subject variability of $C_{p}^{f}$ and $C_{p}^{m}$ in their study. Another contributing factor might be their use of an enzyme‐linked immunosorbent assay that differs from the more specific high‐performance liquid chromatography assay used for most of our data. The peaks of the $C_{p}^{f}/C_{p}^{m}$ versus time curve indicate faster distribution of CSs to the fetus within first few hours after dosing than at later times. Given the absence of the peaks in the reported data, they might be a consequence of not including some processes occurring in the placental transfer of the drug in our mPBPK model. Of importance, our model included the joint permeability and P‐gp–mediated active placental efflux (CL^p^) of the drugs. The CSs are substrates of P‐gp.^35^ Overall, our modeling captured the extensive BET data quite well.

Our modeling did not take into account clearances acting on only free drug, but these CSs are only weakly bound (unbound drug fraction *f*_u_ near 0.35),^4^ have similar blood:plasma ratio values, and the fetal:maternal albumin ratio averages 1.2 ± 0.18 at 35 or more weeks,^22^ all indicating that any adjustment for protein binding would not alter our interpretations of the CS PK data. It has been reported that the presence of proteins actually enhances organic anion transport,^36^ although such an effect on P‐gp transport of the neutral CS is unknown.

We assumed that the ratio of fetal metabolism (CL^f^) to maternal (CL^m^) systemic metabolism was 1:33.8, although CYP3A7 may differ in abundance than CYP3A4 in the neonate (Table S2). These CSs are low‐clearance drugs (adult CL^m^) of DEX = 26.7 L/h compared to (hepatic blood flow *Q*_hepatic_ = 90 L/h) with weak protein binding that partly justifies this assumption and also providing expectation of a small first‐pass effect. Furthermore, an extensive assessment by Upreti and Wahlstrom^37^ indicates that CYP3A activity in newborn infants is only 5%–10% of adult values, with fetal CYP3A functioning thus expected to be even less. A different ratio of CL^f^ to CL^m^ would alter the CL^p^ values proportionally to attain the same fetal AUC values. Furthermore, the GSA indicated that the CL^p^ contributed negligibly to the variability in fetal exposure of both DEX and BET (Figures S4 and S6).

A full, state‐of‐the‐art PBPK model for antenatal CS during pregnancy was recently applied by Anoshchenko et al.^38^ Although highly mechanistic using extensive physiological and PK data for both BET and DEX and including P‐gp placental efflux transport, their modeling did not appear to use and capture well the extensive array of data available in the mother and fetus after dosing BET‐PA (Figure 4) as well as our extensive PK data in nonpregnant Indian women.^27^, ^31^ A smaller absorption rate value for BET‐acetate might rectify this. However, their mean fetal:maternal ratio of BET was only slightly larger (0.48) than ours (0.3–0.4) as generated from a similar ${\mathrm{CL}}^{p}$ and placental blood flow as ours. None of these models (including ours) captures the P‐gp–mediated efflux of DEX from the brain,^29^ and the prediction methods for tissue:plasma ratios in full PBPK models should be considered as only approximate.^28^ Of interest, they report that trans‐well efflux studies of BET and DEX in P‐gp overexpressing Madin‐Darby canine kidney (MDCK) cells revealed similar values, supporting our ${\mathrm{CL}}^{p}$ values, although the latter are also affected by the bidirectional permeability.

The target antenatal CS dosing regimens for DEX and BET should be the lowest dose to maintain maximum therapeutic effects while minimizing CS exposure and potential risks of high maternal and fetal plasma concentrations.^39^, ^40^ We simulated the four antenatal CS regimens recommended by WHO to compare their PK profiles.^12^ Our simulations showed that $C_{\max}^{m}$ values for parturient women were 20%–50% lower than for nonpregnant women receiving CS under the same dosing regimens.^2^ The $\mathrm{AU}C_{0–72}^{m}$ values were 40%–60% lower. This is consistent with the reciprocal relationships between AUC and ${\mathrm{CL}}^{m}$ for these populations. The observed 0.28 ratio for $\mathrm{AU}C_{0–72}^{f}/\mathrm{AU}C_{0–72}^{m}$ is very close to the $C_{p}^{f}/C_{p}^{m}$ ratio at equilibrium.

The GSA of key model parameters for DEX‐P and BET‐P i.m. administration implies that $C_{\max}^{m}$ is mostly influenced by ${\mathrm{CL}}^{m}$ and $k_{\mathrm{aIM}},$ which agrees with the role these parameters play in describing the drug plasma clearance and absorption from the muscle. A second pair of parameters $C_{\max}^{m}$ is most sensitive to is $K_{p}^{m}$ and $V_{t}^{m}$, indicating the importance of the size and composition of the maternal extravascular tissue for the peak maternal plasma concentration of CSs. $C_{\text{trough}}^{m}$ follows a similar pattern except for BET‐P, where ${\mathrm{CL}}^{m}$ is a sole contributor. The peak of fetal plasma concentration $C_{\max}^{f}$ as well as $C_{\text{trough}}^{f}$ and $\mathrm{AU}C_{0–72}^{f}$ are primarily affected by $K_{p}^{f}$, $\;Q_{p}^{m}$, and ${\mathrm{CL}}^{p}$, all placental parameters, confirming the role of the placenta in the fetal PK of CSs. In case of BET‐P, ${\mathrm{CL}}^{m}$ is also an important parameter influencing $C_{\text{trough}}^{f}$ and $\mathrm{AU}C_{0–72}^{f}$. The single most important parameter dominating $\mathrm{AU}C_{0–72}^{m}$ is the maternal plasma clearance ${\mathrm{CL}}^{m}$, which is a consequence of the AUC being equal to dose/clearance. An intriguing absence of the blood volumes $V_{B}^{m}$ and $V_{B}^{f}$ in the list of essential parameters controlling maternal and fetal PK can be explained by a relative character of the Sobol sensitivity indices. Our findings do not imply that these parameters (as well as other low Sobol index parameters) do not contribute to CS PK but, rather, that their contribution to the variability is small compared with other parameters included in GSA.

Although dose/response antenatal CS studies showing an exposure/response relationship are lacking, a suggested target therapeutic efficacy and safety for the use of BET in RDS (based on the data shown in Figure 4) is keeping the fetal plasma concentrations above 1 ng/ml during a 48‐h period.^41^, ^42^ The BET regimens simulated in Figure 5 and summarized in Table 2 achieve this target. The low‐dose regimen of BET‐P 2 mg 4 × 12 h is being tested in the WHO ACTION III trial for the late preterm period.^43^ A major question pertains to the relative efficacy of the DEX exposures. Although the DEX‐P 6 mg 4 × 12 h regimen is efficacious, a lower exposure than BET may be optimal. We reported pharmacodynamic sensitivity values such as the drug plasma concentration eliciting 50% of the maximum inhibition IC_50_ of BET and DEX for cortisol, lymphocytes, neutrophils, and glucose responses in nonpregnant women.^44^ DEX was 1.8 times more potent than BET on average (i.e., lower IC_50_). If the sensitivities of these responses are relevant to efficacy in the fetus (a major assumption), then based on the difference in potency and lower trough DEX concentrations, it would appear that plasma CS exposure following the WHO‐recommended regimens simulated in Figure 5 are consistent for BET and DEX.

In summary, we developed a general hybrid mPBPK model for drugs in pregnant women. The model has been applied to describe the PK of antenatal BET and DEX in parturient women and their fetuses. Although the model was based on maternal and fetal data at the times of previous studies, one can extend it to earlier in pregnancy by using the GA as a covariate for many physiological parameters. Our estimates of maternal clearances of BET and DEX confirmed the values reported from individual studies. The simulations of the fetal‐to‐maternal ratios of plasma concentrations showed similar values for BET and DEX. The simulations of maternal plasma concentrations for antenatal CS dosing regimens recommended by WHO revealed up to 60% lower exposures than in nonpregnant women, but our mPBPK model and previous efficacy studies support current WHO recommendations for dosing both BET and DEX. Our general hybrid mPBPK model and meta‐analysis approach can be applied to other classes of drugs indicated for the treatment of pregnant women.

## AUTHOR CONTRIBUTIONS

W.K., M.A.M., A.H.J., and W.J.J. wrote the manuscript. W.K., M.A.M., A.H.J., and W.J.J. designed the research. W.K., M.A.M., A.H.J., and W.J.J. performed research. W.K. analyzed the data.

## FUNDING INFORMATION

This work was supported by the Bill & Melinda Gates Foundation (Contract No. INV 040110) to Certara Inc and by National Institutes of Health Grant R35 GM131800 to W.J.J.

## CONFLICT OF INTEREST

All authors received financial support from the Bill & Melinda Gates Foundation.

## Supporting information


Appendix S1:

## Data Availability

The data were digitized from the original publications and are provided in their entirety in our graphs.

## References

[1] Abduljalil K , Ning J , Pansari A , Pan X , Jamei M . Prediction of maternal and fetoplacental concentrations of cefazolin, cefuroxime and amoxicillin during pregnancy using bottom‐up physiologically based pharmacokinetic models. Drug Metab Dispos. 2022;50:386‐400. 35046066 10.1124/dmd.121.000711

[2] Liu XI , Momper JD , Rakhmanina NY , et al. Physiologically based pharmacokinetic modeling framework to predict neonatal pharmacokinetics of transplacentally acquired emtricitabine, dolutegravir, and raltegravir. Clin Pharmacokinet. 2021;60:795‐809. 33527213 10.1007/s40262-020-00977-w PMC9334904

[3] Mian P , van den Anker JN , van Calsteren K , et al. Physiologically based pharmacokinetic modeling to characterize acetaminophen pharmacokinetics and N‐acetyl‐p‐benzoquinone imine (NAPQI) formation in non‐pregnant and pregnant women. Clin Pharmacokinet. 2020;59:97‐110. 31347013 10.1007/s40262-019-00799-5 PMC6994454

[4] Ke AB , Milad MA . Evaluation of maternal drug exposure following the administration of antenatal corticosteroids during late pregnancy using physiologically‐based pharmacokinetic modeling. Clin Pharmaco Ther. 2019;106:164‐173. 10.1002/cpt.1438 30924921

[5] Pillai VC , Shah M , Rytting E , et al. Prediction of maternal and fetal pharmacokinetics of indomethacin in pregnancy. Br J Clin Pharmacol. 2022;88:271‐281. 34185331 10.1111/bcp.14960

[6] Chaphekar N , Caritis S , Venkataramanan R . Model‐informed dose optimization in pregnancy. J Clin Pharmacol. 2020;60(Suppl 1):S63‐S76. 10.1002/jcph.1777 33205432

[7] Nestorov IA , Aarons LJ , Arundel PA , Rowland M . Lumping of whole‐body physiologically based pharmacokinetic models. J Pharmacokinet Biopharm. 1998;26:21‐46. 9773391 10.1023/a:1023272707390

[8] Cao Y , Jusko WJ . Applications of minimal physiologically‐based pharmacokinetic models. J Pharmacokinet Pharmacodyn. 2012;39:711‐723. 23179857 10.1007/s10928-012-9280-2 PMC3539784

[9] Song D , Jusko WJ . Across‐species meta‐analysis of dexamethasone pharmacokinetics utilizing allometric and scaling modeling approaches. Biopharm Drug Dispos. 2021;42:191‐203. 33638217 10.1002/bdd.2266 PMC8859844

[10] Committee opinion No. 677. ACOG committee opinion: antenatal corticosteroid therapy for fetal maturation. Obstet Gynecol. 2016;128:e187‐e194. 27661658 10.1097/AOG.0000000000001715

[11] Raikkonen K , Gissler M , Kajantie E . Associations between maternal antenatal corticosteroid treatment and mental and behavioral disorders in children. JAMA. 2020;323:1924‐1933. 32427304 10.1001/jama.2020.3937 PMC7237984

[12] World Health Organization (WHO) . Recommendations on Interventions to Improve Preterm Birth Outcomes. WHO; 2015. 26447264

[13] Szeto KX , Merdy ML , Dupont B , Bolger MB , Lukacova V . PBPK modeling approach to predict the behavior of drugs cleared by kidney in pregnant subjects and fetus. AAPS J. 2021;23:89. 34169370 10.1208/s12248-021-00603-y PMC8225528

[14] Lederman SA , Pierson RN Jr , Wang J , et al. Body composition measurements during pregnancy. Basic Life Sci. 1993;60:193‐195. 8110108 10.1007/978-1-4899-1268-8_44

[15] Stevens JC , Hines RN , Gu C , et al. Developmental expression of the major human hepatic CYP3A enzymes. J Pharmacol Exp Ther. 2003;307:573‐582. 12975492 10.1124/jpet.103.054841

[16] Sala L , Golse N , Joosten A , Vibert E , Vignon‐Clementel I . Sensitivity analysis of a mathematical model simulating the post‐hepactomy hemodynamics response. Ann Biomed Eng. 2022. doi:10.1007/s10439-022-03098-6 PMC9832106 36326994

[17] Bloomingdale P , Bakshi S , Maass C , et al. Minimal brain PBPK model to support the preclinical and clinical development of antibody therapeutics for CNS diseases. J Pharmacokinet Pharmacodyn. 2021;48:861‐871. 34378151 10.1007/s10928-021-09776-7 PMC8604880

[18] Li X , Jusko WJ , Cao Y . Role of interstitial fluid turnover on target suppression by therapeutic biologics using a minimal physiologically based pharmacokinetic model. J Pharmacol Exp Ther. 2018;367:1‐8. 30002096 10.1124/jpet.118.250134 PMC6123664

[19] Scotcher D , Jones C , Rostami‐Hodjegan A , Galetin A . Novel minimal physiologically‐based model for the prediction of passive tubular reabsorption and renal excretion clearance. Eur J Pharm Sci. 2016;94:59‐71. 27033147 10.1016/j.ejps.2016.03.018 PMC5074076

[20] Maas BM , Cao Y . A minimal physiologically based pharmacokinetic model to investigate FcRn‐mediated monoclonal antibody salvage: effects of K(on), K(off), endosome trafficking, and animal species. MAbs. 2018;10:1322‐1331. 30130450 10.1080/19420862.2018.1506648 PMC6284604

[21] Xia B , Heimbach T , Gollen R , Nanavati N , He H . A simplified PBPK modeling approach for prediction of pharmacokinetics of four primarily renally excreted and CYP3A metabolized compounds during pregnancy. AAPS J. 2013;15:1012‐1023. 23835676 10.1208/s12248-013-9505-3 PMC3787241

[22] Abduljalil K , Jamel M , Johnson TN . Fetal physiologically based pharmacokinetic models: systems information on fetal blood components and binding proteins. Clin Pharmacokin. 2020;59:629‐642. 10.1007/s40262-019-00836-3 31696406

[23] Goetzl L , Darbinian N , Merabova N , Devane LC , Ramamorthy S . Gestational age variation in human placental drug transporters. Front Pharmacol. 2022;13: Article 837694. 10.3389/fphar.2022.837694 PMC9019509 35462922

[24] McCune JS , Hawke RL , LeCluyse EL , et al. In vivo and in vitro induction of humancytochrome P4503A4 by dexamethasone. Clin Pharmacol Ther. 2000;68:356‐366. 11061575 10.1067/mcp.2000.110215

[25] Tsuei SE , Petersen MC , Ashley JJ , McBride WG , Moore RG . Disposition of synthetic glucocorticoids II. Dexamethasone in parturient women. Clin Pharmacol Ther. 1980;28:88‐98. 7389259 10.1038/clpt.1980.136

[26] Petersen MC , Collier CB , Ashley JJ , McBride WG , Nation RL . Disposition of betamethasone in parturient women after intravenous administration. Eur J Clin Pharmacol. 1983;25:803‐810. 6662178 10.1007/BF00542524

[27] Jobe AH , Milad MA , Peppard T , Jusko WJ . Pharmacokinetics and pharmacodynamics of intramuscular and oral betamethasone and dexamethasone in reproductive age women in India. Clin Transl Sci. 2020;13:391‐399. 31808984 10.1111/cts.12724 PMC7070803

[28] Giaginis C , Zira A , Theocharis S , Tsantili‐Kakoulidou A . Application of quantitative structure=activity relationships for modeling drug and chemical transport across the human placenta barrier: a multivariate data analysis approach. J Appl Toxicol. 2009;29:724‐733. 19728316 10.1002/jat.1466

[29] Song D , Sun L , DuBois DC , Almon RR , Meng S , Jusko WJ . Physiologically‐based pharmacokinetics of dexamethasone in rats. Drug Metab Disp. 2020;48:811‐818. 10.1124/dmd.120.091017 PMC7448200 32601175

[30] Petersen MC , Ashley JJ , McBride WG , Nation RL . Disposition of betamethasone in parturient women after intramuscular administration. Br J Clin Pharmacol. 1984;18:383‐392. 6487477 10.1111/j.1365-2125.1984.tb02480.x PMC1463657

[31] Krzyzanski W , Milad MA , Jobe AH , Peppard T , Bies RR , Jusko WJ . Population pharmacokinetic modeling of intramuscular and oral dexamethasone and betamethasone in Indian women. J Pharmacokinet Pharmacodyn. 2021;48:261‐272. 33389521 10.1007/s10928-020-09730-z PMC7778726

[32] Petersen MC , Nation RL , Ashley JJ , McBride WG . The placental transfer of betamethasone. Eur J Clin Pharmacol. 1980;18:245‐247. 7439243 10.1007/BF00563006

[33] Ballard PL , Granberg P , Ballard RA . Glucocorticoid levels in maternal and cord serum after prenatal betamethasone therapy to prevent respiratory distress syndrome. J Clin Invest. 1975;56:1548‐1554. 1202085 10.1172/JCI108236 PMC333133

[34] Zafran N , Massalha M , Suleiman A , et al. Association between betamethasone levels and respiratory distress syndrome in preterm births: a prospective cohort study. Clin Transl Sci. 2022;15:2528‐2537. 35923139 10.1111/cts.13382 PMC9579395

[35] Yates CR , Chang C , Kearbey JD , et al. Structural determinants of P‐glycoprotein‐mediated transport of glucocorticoids. Pharm Res. 2003;20:1794‐1803. 14661924 10.1023/b:pham.0000003377.39548.f6

[36] Yin M , Storelli F , Unadkat JD . Is the protein‐mediated uptake of drugs by organic anion transporting polypeptides a real phenomenon or an artifact? Drug Metab Dispos. 2022;50:1132‐1141. 35351775 10.1124/dmd.122.000849

[37] Upreti VV , Wahlstrom JL . Meta‐analysis of hepatic cytochrome P450 ontogeny to underwrite the prediction of pediatric pharmacokinetics using physiologically based pharmacokinetic modeling. J Clin Pharmacol. 2016;56:266‐283. doi:10.1002/jcph.585 26139104

[38] Anoshchenko O , Milad MA , Unadkat JD . Estimating fetal exposure to the P‐gp substrates, corticosteroids, by PBPK modeling to inform prevention of neonatal respiratory distress syndrome. CPT:PSP. 2021;10:1057‐1070. 34273255 10.1002/psp4.12674 PMC8452292

[39] Jobe AH , Goldenberg RL . Antenatal corticosteroids: an assessment of anticipated benefits and potential risks. Am J Obst Gyn. 2018;219(1):62‐74. 10.1016/j.ajog.2018.04.007 29630886

[40] Ninan K , Liyanage SK , Murphy KF , Asztakos EV , McDonald SD . Evaluation of long‐term outcomes associated with preterm exposure to antenatal corticosteroids. A systematic review and meta‐analysis. JAMA Pediat. 2022;176(6):e220483. 10.1001/jamapediatrics.2022.0483 PMC9002717 35404395

[41] Kemp MW , Saito M , Usuda H , et al. The efficacy of antenatal steroid therapy is dependent on the duration of low‐concentration fetal exposure: evidence from a sheep model of pregnancy. Am J Obst Gyn. 2018;301:E1‐E16. 10.1016/j.ajog.2018.05.007 29758177

[42] Foissac F , Zheng Y , Hirt D , et al. Maternal betamethasone for prevention of respiratory distress syndrome in neonates: population pharmacokinetic and pharmacodynamic approach. Clin Pharmacol Ther. 2020;108:1026‐1035. 32394434 10.1002/cpt.1887

[43] WHO . ACTION III: A multi‐country, multi‐centre, three‐arm, parallel group, double‐blind, placebo‐controlled, randomized trial of two doses of antenatal corticosteroids for women with a high probability of birth in the late preterm period in hospitals in low‐resource countries to improve newborn outcomes, Feb 25, 2021.

[44] Krzyzanski W , Milad MA , Jobe AH , Peppard T , Bies RR , Jusko WJ . Population pharmacodynamic modeling of intramuscular and oral dexamethasone and betamethasone effects on six biomarkers with circadian complexities in Indian women. J Pharmacokinet Pharmacodyn. 2021;48:411‐438. 33954911 10.1007/s10928-021-09755-y PMC8099395

