# Supplementary material for: Minimal physiologically‐based hybrid model of pharmacokinetics in pregnant women: Application to antenatal corticosteroids
Source: CPT Pharmacometrics Syst Pharmacol. 2023 Mar 14;12(5):668–80. doi: 10.1002/psp4.12899 (PMC10196440; doi:10.1002/psp4.12899)
Supplement: Supplementary file 1 — Appendix S1: [file PSP4-12-668-s001.pdf]

Supplementary Materials

Minimal physiologically based hybrid model of drug pharmacokinetics in pregnant women. Application to antenatal corticosteroids.

Wojciech Krzyzanski, Mark A Milad, Alan H Jobe, William J Jusko

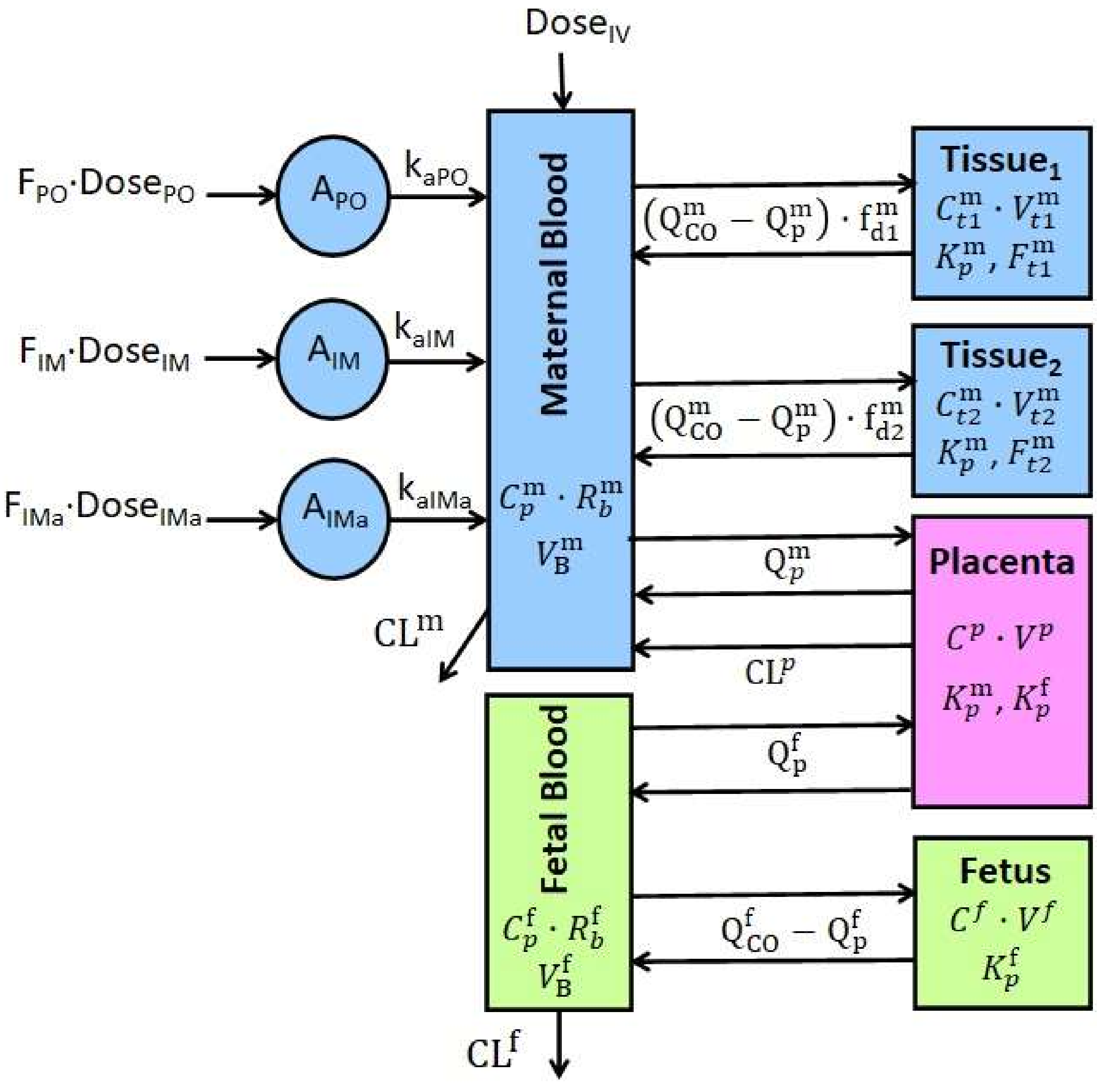

Fig S1. Diagram for the mPBPK model for antenatal corticosteroids DEX and BET that includes the dosing regimens. Model parameters are defined in Tables 1 and 2S

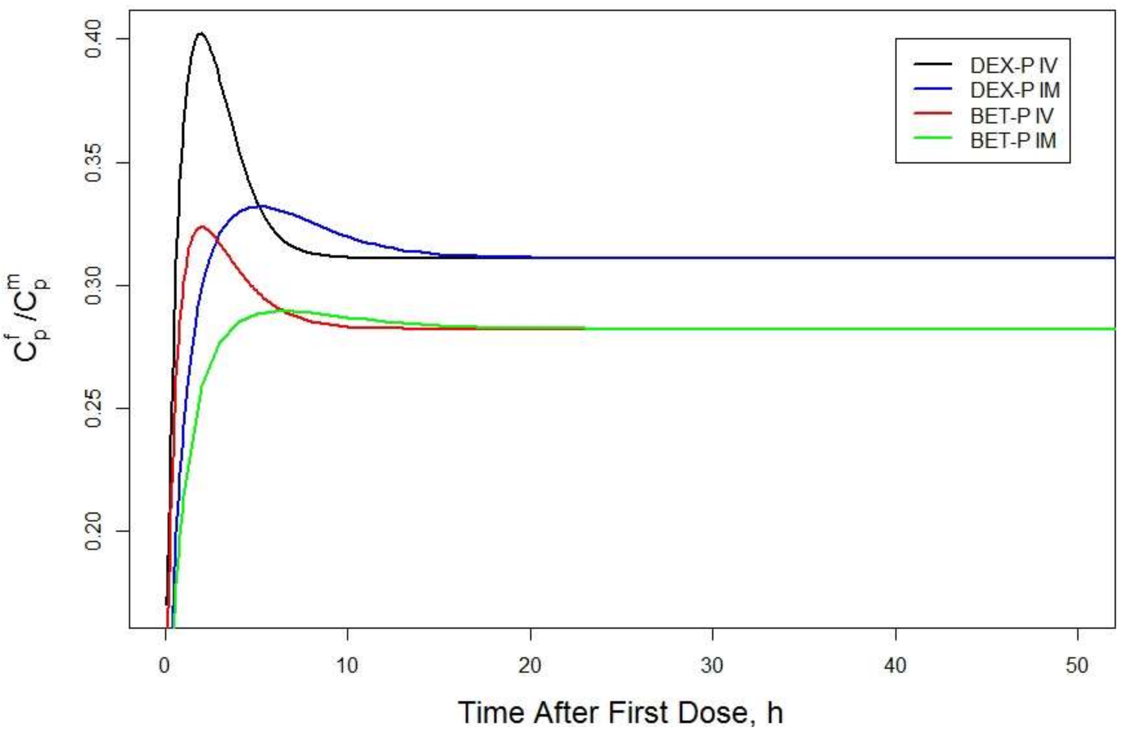

Fig. S2. Simulated fetal to maternal plasma concentration ratios for indicated 8 mg single doses of DEX-P and BET-P administered IV and IM.

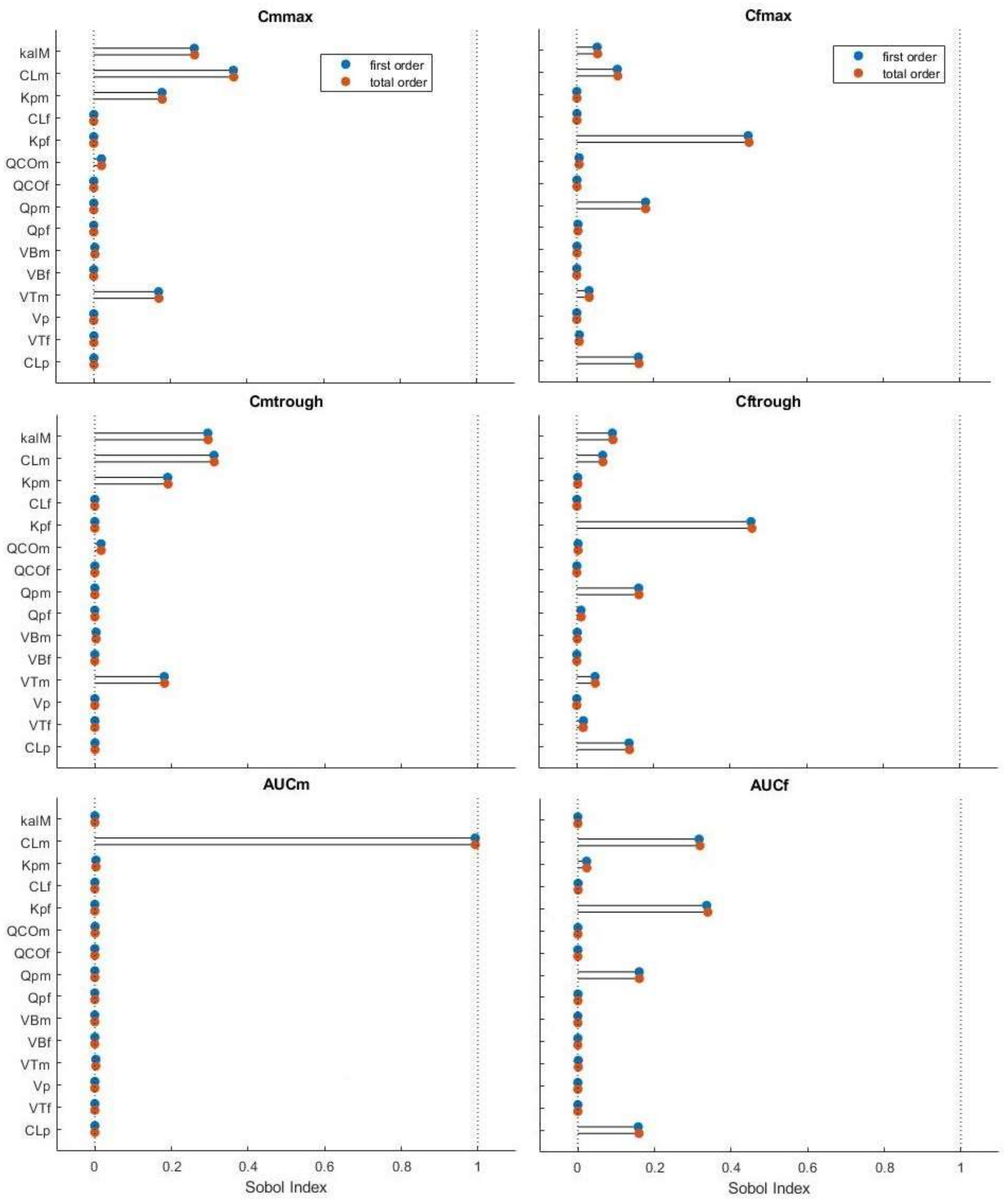

Fig. S3. Sobol first-order and total-order indices for the indicated parameters obtained for the outputs C^{m}_{max}, C^{f}_{max}, C^{m}_{trough}, C^{f}_{trough}, AUC^{m}_{0-72}, and AUC^{f}_{0-72}, corresponding to the DEX-P IM 6 mg 4x12h dosing regimen.

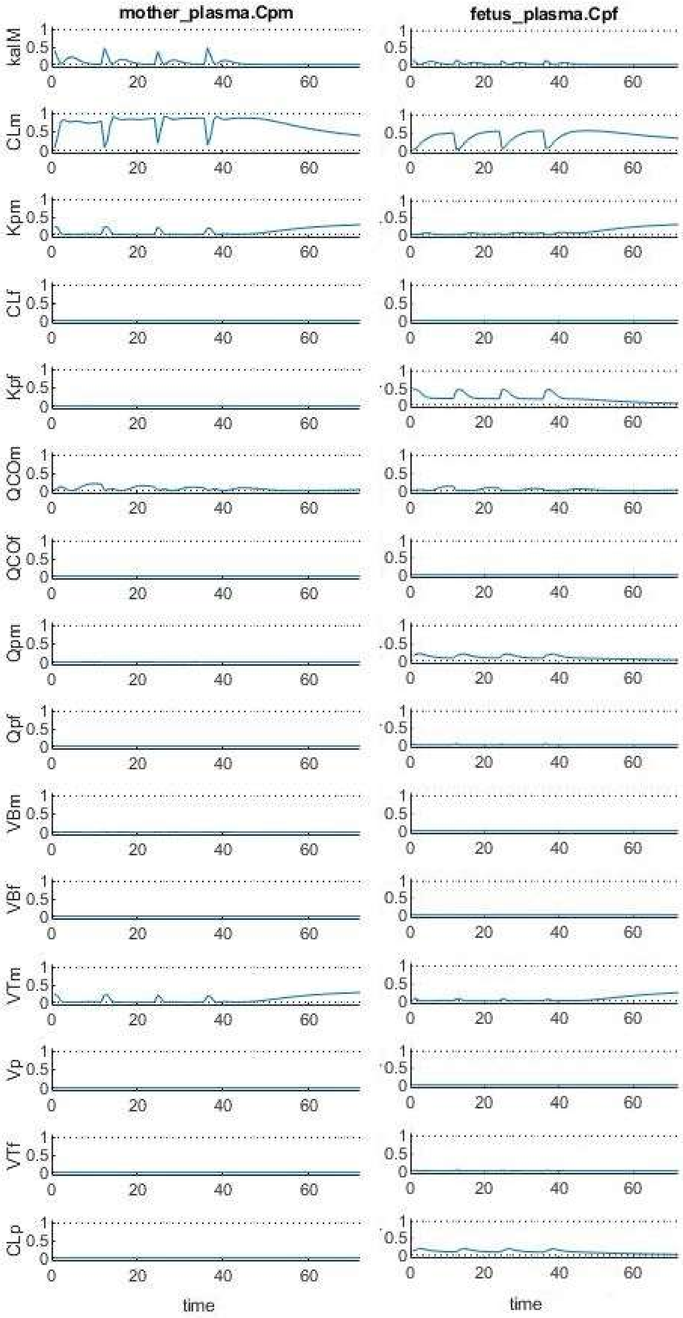

Fig. S4. Time courses of the Sobol first-order indices for the indicated parameters obtained for the maternal and fetal plasma concentrations C_p^m and C_p^f of DEX following the DEX-P IM 6 mg 4x12 h dosing regimen.

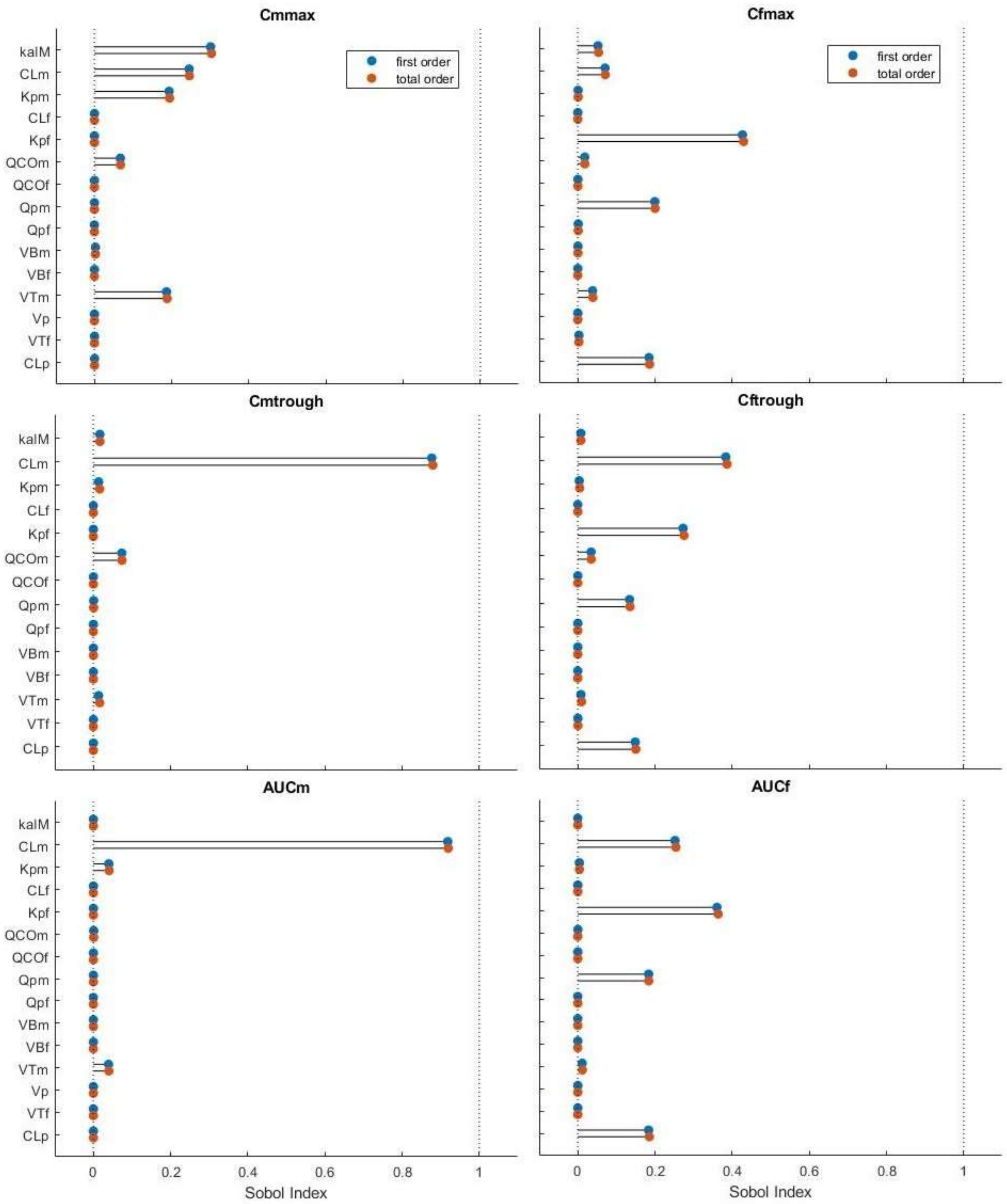

Fig. S5. Sobol first-order and total-order indices for the indicated parameters obtained for the outputs C^{m}_{max}, C^{f}_{max}, C^{m}_{trough}, C^{f}_{trough}, AUC^{m}_{0-72}, and AUC^{f}_{0-7} , corresponding to the BET-P IM 12 mg 2x24h dosing regimen.

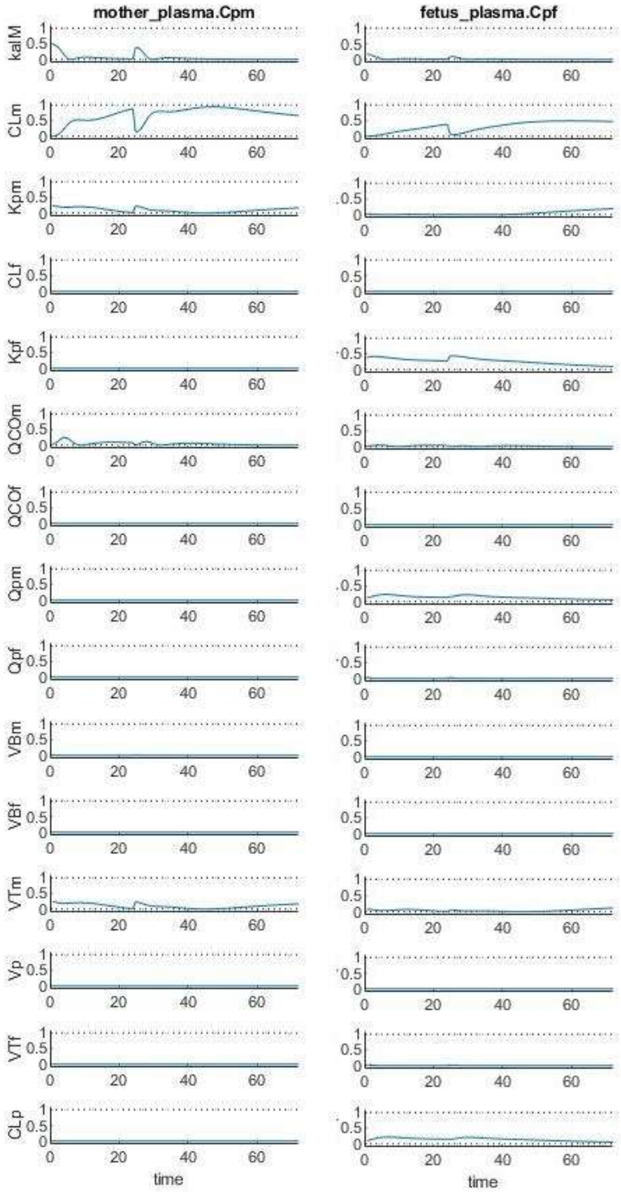

Fig. S6. Time courses of the Sobol first-order indices for the indicated parameters obtained for the maternal and fetal plasma concentrations C_p^m and C_p^f of BET following the BET-P IM 12 mg 2x24 h dosing regimen.

Table S1. Published articles reporting DEX and BET plasma concentrations in pregnant women.

Source	Drug	Route	Dosing	Data
Tsuei et al.[1]	DEX-P	IM	8 mg 2x24h	MPV, UV
Tsuei et al.[1]	DEX-P	IV	8 mg	MPV
Kream et al.[2]	DEX-P	IM	5 mg 4x12h	MPV
Osathanondh et al.[3]	DEX-P	PO	8 mg	MPV, UV
Egerman et al.[4]	DEX-P	IM, PO	6, 8 mg	MPV
Petersen et al.[5]	BET-P	IV, IM	8 mg 2x24h	MPV, UV
Petersen et al.[6]	BET-P	IV	8 mg	MPV
Petersen et al.[7]	BET-P	IM	8 mg	MPV
Ballard et al.[8]	BET-PA	IM	12 mg 2x24h	MPV, UV
Ballabh et al.[9]	BET-PA	IM	12.5 2x24h	MPV, UV
Gyamfi et al.[10]	BET-PA	IM	12 mg 2x24h	MPV, UV
Suvari et al.[11]	BET-PA	IM	12 mg 2x24h, 2x12h	UV
Foissac et al.[12]	BET-PA	IM	11.4 2x24 h	MPV, UV

DEX-P = dexamethasone phosphate

BET-P = betamethasone phosphate

BET-PA = 1:1 mixture of betamethasone phosphate and acetate

PO = oral administration

IM = intramuscular injection

IV = intravenous injection

MPV = maternal peripheral vein

UV = umbilical vein

Table S2. Definitions and values of the mPBPK model parameters for DEX and BET in pregnant women. The parameter values were obtained from literature, assigned, or calculated from the parameters estimated from fitting the data. Estimates of remaining model parameters are listed in Table 1.

Parameter	Definition	Value	Source
K_p^f	Fetal tissue to plasma partition coefficient	2.37	$K_p^f = K_p^m$
R_b^m	Maternal blood to plasma ratio	1.0	Assigned
R_b^f	Fetal blood to plasma ratio	1.0	Assigned
f_{d2}^m	Fraction of cardiac output for $Tissue_2$	0.047	$f_{d2}^m = 1 - f_{d1}^m$
F_{t2}^m	Fraction of total tissue volume for $Tissue_2$	0.766	$F_{t2}^m = 1 - F_{t1}^m$
Q_{CO}^m, L/h	Maternal cardiac output at GA = 39 wk	397.9	Abduljalil et al.[13]
Q_{CO}^f, L/h	Fetal cardiac output at GA=39 wk	82.62	Mielke et al.[14]
Q_p^m, L/h	Maternal blood flow in placenta at GA=39 wk	39.0	Wang et al.[15]
Q_p^f, L/h	Blood flow in umbilical cord at GA=29 wk	26.58	Wang et al.[15]
CL_{DEX}^f, L/h	DEX fetal blood clearance	0.790	$CL_{DEX}^f = CL_{DEX}^m/33.8$*
CL_{BET}^f, L/h	BET fetal blood clearance	0.264	$CL_{BET}^f = CL_{BET}^m/33.8$*
BW^m, kg	Maternal body weight at GA = 39 wk	76.27	Abduljalil et al.[13]
ρ, kg/L	Tissue density	1.0	Assigned
V^f, L	Fetal volume at GA=39 wk	3.356	Abduljalil et al.[13]
V^p, L	Volume of placenta at GA=39 wk	0.640	Abduljalil et al.[13]
V_a, L	Volume of amniotic fluid at GA = 39 wk	0.886	Abduljalil et al.[13]
Q_{CO}^f, L/h	Fetal cardiac output at GA=39 wk	82.62	Mielke et al.[14]
Q_p^m, L/h	Maternal blood flow in placenta at GA=39 wk	39.0	Wang et al.[15]
Q_p^f, L/h	Blood flow in umbilical cord at GA=29 wk	26.58	Wang et al.[15]
V_B^m, L	Maternal blood volume at GA=39 wk	5.738	Abduljalil et al.[13]
V_B^f, L	Fetoplacental blood volume at GA=39 wk	0.471	Mandelbrot et al.[16]
V_t^m, L	Volume of maternal tissue	65.66	$V_t^m = BW^m/\rho - V_B^m - V^f - V^p - V_a$
V_t^f, L	Volume of fetal tissue	2.96	$V_t^f = V^f - V_B^f \cdot V^f/(V^f + V^p)$
V_{t1}^m, L	Volume of maternal $Tissue_1$	15.36	$V_{t1}^m = F_{t1}^m \cdot V_t^m$
V_{t2}^m, L	Volume of maternal $Tissue_2$	50.29	$V_{t2}^m = F_{t2}^m \cdot V_t^m$

*CL^f is calculated as a fraction of CL^m based on a 33.8-fold greater intrinsic clearance of 7-hydroxy-dehydroepiandrosterone by CYP3A4 in mother versus CYP3A7 expression in fetus.[17]

REFERENCES

1. Tsuei, S.E., Petersen, M.C., Ashley, J.J., McBride, W.G., Moore, R.G. Disposition of synthetic glucocorticoids II. Dexamethasone in parturient women. *Clin Pharmacol Ther* **28**, 88-98 (1980).

2. Kream, J., Mulay, S., Fukushima, D.K., Solomon, S. Determination of plasma dexamethasone in the mother and the newborn after administration of the hormone in a clinical trial. *J Clin Endocrinol Metab* **56**, 127-133 (1983).

3. Osathanondh, R., Tulchinsky, D., Kamali, H., Fencl, M.D., Taeusch, H.W. Dexamethasone levels in treated pregnant women and newborn infants. *J Pediatr* **90**, 617-620 (1977).

4. Egerman, R.S., Pierce, W.F., Andersen, R.N., Umstot, E.S., Carr, T.L., Sibai, B.M. A comparison of the bioavailability of oral and intramuscular dexamethasone in women in late pregnancy. *Obst & Gynecol* **89**, 276-280 (1997).

5. Petersen, M.C., Nation, R.L., Ashley, J.J., McBride, W.G. The placental transfer of betamethasone. *Eur J Clin Pharmacol* **18**, 245–247 (1980).

6. Petersen, M.C., Collier, C.B., Ashley, J.J., McBride, W.G., Nation, R.L. Disposition of betamethasone in parturient women after intravenous administration. *Eur J Cli. Pharmacol* **25**, 803-810 (1983).

7. Petersen, M.C., Ashley, J.J., McBride, W.G., Nation, R.L. Disposition of betamethasone in parturient women after intramuscular administration. *Br J Clin Pharmacol* **18**, 383-392 (1984).

8. Ballard, P.L., Granberg, P., Ballard, R.A. Glucocorticoid levels in maternal and cord serum after prenatal betamethasone therapy to prevent respiratory distress syndrome. *J Clin Invest* **56**, 1548-1554 (1975).

9. Ballabh, P., Lo, E.S., Kumari, J., Cooper, T.B., Zervoudakis, I., Auld, P.A.M., Krauss, A.N. Pharmacokinetics of betamethasone in twin and singleton pregnancy. *Clin Pharmacol Ther* **71**, 39-45 (2002).

10. Gyamfi, C., Mele, L., Wapner, R.J., Spong, C.Y., Peaceman, A., Sorokin, Y., Dudley, D.J., Johnson, F., Leveno, K.J., Caritis, S.N., Mercer, B.M., Thorp, J.M., O'Sullivan, M.J., Ramin, S.M., Carpenter, M., Rouse, D.J., Miodovnik, M., Sibai, B. (2010) The effect of plurality and obesity on betamethasone concentrations in women at risk for preterm delivery. *Am J Obstet Gynecol* **203**, 219.e1-5 (2010).

11. Suvari, L., Helve, O.M., Kari, M.A., Turpeinen, L.U., Palojärvi, P.A., Leskinen, M.J., Andersson, S., Janér, A.C. Glucocorticoids, sodium transport mediators, and respiratory distress syndrome in preterm infants. *Ped Res* **14**, 1-8 (2020).

12. Foissac, F., Zheng, Y., Hirt, D., Lui, G., Bouazza, N., Ville, Y., Goffinet, F., Rozenberg, P., Kayem, G., Mandelbrot, L., Benaboud, S., Jarreau, P.H., Tréluyer, J.M. Maternal betamethasone for prevention of respiratory distress syndrome in neonates: population pharmacokinetic and pharmacodynamic approach. *Clin Pharmacol Ther* **108**, 1026-1035 (2020).

13. Abduljalil, K., Furness, P., Johnson, T.N., Rostami-Hodjegan, A., Soltani, H. Anatomical, physiological and metabolic changes with gestational age during normal pregnancy. A database for parameters required in physiologically based pharmacokinetic modelling. *Clin Pharmacokinet* **51,** 365-396 (2012).

14. Mielke, G., Benda, N. Cardiac output and central distribution of blood flow in the human fetus. *Circulation* **103**,1662-1668 (2001).

15. Wang, Y., Zhao, S. Vascular Biology of the Placenta. Morgan & Claypool Life Sciences, San Rafael (CA) (2010).

16. Mandelbrot, L., Daffos, F., Forestier, F., MacAleese, J., Descombey, D. Assessment of fetal blood volume for computer-assisted management of in utero transfusion. *Fetal Ther* **3**, 60-66 (1998).

17. Stevens, J.C., Hines, R.N., Gu C., Koukouritaki, S.B., Manro, J.R., Tandler, P.J., Zaya, M.J. Developmental expression of the major human hepatic CYP3A enzymes. *J Pharmacol Exp Ther* **307**, 573–582 (2003).
